# Geopolymer, Alkali-Activated, and Waste-Derived Binders for Soft-Soil Stabilization: A Critical Review

**DOI:** 10.3390/ma19214546

**Published:** 2026-10-28

**Authors:** Rui Jia, Kejin Jiang, Zhuo Li, Xinyue Wang

**Affiliations:** 1 School of Civil Engineering, Tianjin University, 135 Yaguan Road, Jinnan District, Tianjin 300354, China; lizhuo2421@163.com; 2 Key Laboratory of Coast Civil Structure Safety of the Ministry of Education, Tianjin University, 135 Yaguan Road, Jinnan District, Tianjin 300354, China; jkj@tju.edu.cn

**Keywords:** soft-soil stabilization, geopolymer, alkali-activated binder, waste-derived binder, durability, sustainability

## Abstract

Geopolymer, alkali-activated, and waste-derived binders offer alternatives to cement for soft-soil stabilization, but their benefits depend on material and site conditions. This review distinguishes four overlapping groups by dominant reaction chemistry: low-calcium geopolymers, high-calcium alkali-activated binders, hybrid systems, and waste-derived cementitious binders. It separates direct soft-soil evidence from supporting studies on other soils and non-soil binders, linking reaction products, soil–binder interactions, and pore evolution to engineering behavior. In one soft soil with 50.4% initial water content and 20% precursor addition by dry soil mass, 28-day unconfined compressive strengths ranged from 0.09 to 8.22 MPa across fly ash–slag formulations cured at 23 °C. Stabilization generally increases strength and stiffness and reduces compressibility, but stronger cementation can increase brittleness and post-peak softening. Hydraulic conductivity depends on pore connectivity, cracking, and stress. Fly ash–slag blends can balance early strength and workability, but optimum proportions remain soil-specific. Of 74 mapped publications, 63 report laboratory unconfined compression and two report cyclic/dynamic response; creep, coupled durability, and long-term field performance remain poorly documented. Activator production and precursor processing can offset environmental gains from waste reuse. Comparisons with cement require equivalent engineering performance, consistent life-cycle boundaries, and leaching assessment. These findings support soil-specific and performance-based binder design.

## 1. Introduction

### 1.1. Soft-Soil Challenges and Conventional Stabilization

Soft soils are widespread in coastal plains, river deltas, lake basins, reclaimed land, and areas affected by excavation or dredging. They typically have high natural water content and void ratio, low undrained shear strength, high compressibility, low permeability, pronounced sensitivity, and time-dependent deformation. These properties can lead to excessive settlement, bearing-capacity failure, lateral deformation, instability during staged construction, and long consolidation periods. Dredged soils and excavated muds present an additional challenge because their high water content and poor workability make direct reuse difficult [1,2,3,4]. Organic soft soils are particularly difficult to stabilize because humic substances can adsorb reactive ions, buffer alkalinity, interfere with soil–binder bonding, and retard the formation of cementitious products [5,6,7]. Sulfate-rich soils and sulfate exposure can also cause expansive reactions or long-term deterioration in calcium-bearing systems [8,9].

Chemical stabilization is widely used because it can be incorporated into shallow mixing, deep soil mixing, mass stabilization, and the production of engineered fill. Portland cement and lime remain the conventional binders because their design methods, construction procedures, and quality-control practices are well established. Cement production is energy- and carbon-intensive and requires substantial use of virgin mineral resources. For soft-ground treatment, binder efficiency depends strongly on soil conditions. High water content dilutes reactive species, while organic matter and sulfate-rich pore fluids can interfere with cementitious reactions. As a result, slurry-like or chemically challenging soils may require relatively high binder dosages to meet strength and deformation requirements, increasing both cost and embodied carbon [6,10,11,12].

These limitations have encouraged the use of industrial by-products and other alternative binders. Their environmental advantage, however, should not be assumed simply because Portland cement is partly or fully replaced. Life-cycle impacts depend on precursor source and transport, activator type and dosage, thermal or mechanical pretreatment, curing energy, and the amount of binder required to achieve the target engineering performance. In particular, commercial sodium silicate and sodium hydroxide (NaOH) can contribute substantially to the environmental burden of alkali-activated systems [13,14]. One production-stage assessment found similar climate-change impacts for volcanic-ash geopolymer and cement stabilization at comparable shear strength, although other impact categories differed [15]. The key criterion is whether a binder can provide the required engineering function with lower overall environmental and economic cost. Accordingly, these alternative binders should be evaluated on a performance-equivalent basis, considering strength, deformation, durability, service conditions, and life-cycle impacts together [13,15,16].

### 1.2. Geopolymer and Alkali-Activated Binders for Soil Stabilization

Geopolymers are inorganic binders formed by the alkaline dissolution of reactive Si- and Al-bearing solids, followed by reorganization, gel formation, and polycondensation. Foundational studies showed that low-calcium fly ash, metakaolin, and other aluminosilicate sources can form amorphous alkali-aluminosilicate networks under alkaline activation [17,18,19,20]. These binders can develop high strength and chemical resistance while hardening at relatively low temperatures. Their use has therefore expanded from pastes, mortars, and concrete to waste immobilization and, increasingly, geotechnical stabilization [11,21,22,23,24,25,26].

The term “geopolymer,” however, is used rather broadly in the soil-stabilization literature. Low-calcium fly ash and metakaolin systems commonly form N-A-S-H-rich gels and are consistent with the conventional geopolymer concept. By contrast, ground-granulated blast-furnace slag (GGBFS), high-calcium fly ash, and fly ash–slag blends often form C-(A)-S-H, sometimes alongside alkali-aluminosilicate products. Other systems containing calcium carbide residue, soda residue, steel slag, gypsum, plant ash, or silica fume may gain strength mainly through hydration, pozzolanic reactions, sulfate reactions, or carbonation [7,27,28,29]. These materials can be effective alternative stabilizers, but their chemistry should not automatically be described as geopolymerization.

This distinction has practical consequences. Low-calcium systems generally require sufficient alkalinity to dissolve the aluminosilicate precursor, and their early strength development at ambient temperature can be relatively slow. Calcium-rich systems usually react faster and can provide higher early strength, but rapid setting, workability loss, and shrinkage may become more important. Fly ash–slag blends often provide a useful compromise: slag promotes early reaction and calcium-rich gel formation, whereas fly ash can improve workability, moderate setting, and contribute to later aluminosilicate reaction [30,31,32,33]. The optimum balance depends on soil water content, precursor reactivity, activator chemistry, curing conditions, and the required engineering function. A higher slag content is therefore not universally beneficial.

Another important development is the move from conventional two-part systems toward one-part, “just-add-water” binders. Two-part systems use a solid precursor together with NaOH solution, sodium silicate solution, or a combined alkaline solution. They are convenient for laboratory optimization but create practical concerns related to liquid transport, storage, viscosity, corrosion, and operator safety. One-part systems instead premix the precursor with a solid activator for mixing with water or wet soil during use. Solid sodium metasilicate- and sodium silicate-activated systems have shown good potential for soft-clay stabilization and may be more compatible with field mixing [34,35]. Their practical advantages should nevertheless not be overstated. Moisture uptake and caking during storage, alkaline dust, non-uniform activator dissolution, rapid setting in calcium-rich mixtures, and mixture-water control remain important issues. These differences in chemistry and preparation route form the basis for the terminology and binder classification adopted in this review.

### 1.3. Scope, Terminology, and Evidence

#### 1.3.1. Soft Soils and Evidence Boundaries

The core evidence reviewed here focuses on natural marine, coastal, alluvial, and lacustrine soft clays; dredged clay and marine silt; excavated mud and slurry; reconstituted or model soft clay; and organic soft clay. These materials commonly have high water content, weak particle contacts, low stiffness, and large deformation potential. Related problematic soils, including expansive clay, black cotton soil, sulfate-rich clay, loess, residual and weathered soils, and clayey subgrade soil, are used as supporting evidence where they help clarify mineralogical effects, swelling control, durability, or construction performance [9,36,37,38,39].

Studies on geopolymer and alkali-activated pastes, mortars, concrete, mine backfill, and other non-soil materials are considered only when they provide useful evidence on precursor reactivity, activator chemistry, setting, shrinkage, reaction products, waste-derived activation, or life-cycle impacts [14,40,41,42,43,44,45,46]. Their mechanical performance is not transferred directly to stabilized soft soils. Soil introduces additional effects from water dilution, mineral surfaces, organic matter, salinity, pore-fluid chemistry, and stress-dependent fabric changes. These differences are particularly important when interpreting strength, durability, and field applicability.

#### 1.3.2. Binder Terminology and Classification

To avoid treating chemically different binders as equivalent, this review uses four overlapping working categories. Low-calcium geopolymers are based mainly on low-calcium fly ash, metakaolin, calcined clay, or similar aluminosilicate precursors and are dominated by alkali-aluminosilicate gels, commonly described as N-A-S-H or K-A-S-H. High-calcium alkali-activated binders are based mainly on GGBFS, high-calcium fly ash, or other calcium-rich precursors and are dominated by C-(A)-S-H and related calcium-rich products. Hybrid systems, including some fly ash–slag blends, may involve both alkali-aluminosilicate and calcium-rich reaction products. Waste-derived cementitious binders include binders containing calcium carbide residue, soda residue, steel slag, silica fume, rice husk ash, gypsum, and related wastes, for which hydration, pozzolanic reactions, sulfate reactions, or carbonation may contribute substantially to hardening [7,27,29,47,48,49]. These systems are included because they are widely studied as alternative binders for soft-soil stabilization, although their dominant reactions may differ from geopolymerization. Waste origin alone does not define reaction chemistry. One-part and two-part terms describe preparation routes within these categories. For individual formulations, classification considers precursor composition and reactivity, activator chemistry, curing conditions, and available reaction-product evidence. Where available, complementary product evidence takes precedence over precursor labels or bulk calcium content; no universal calcium-content threshold is applied. Evidence supporting both alkali-aluminosilicate and calcium-rich gels supports a hybrid interpretation, without establishing their relative contributions or a single homogeneous gel. Where product identification is inconclusive, the classification remains provisional.

Reaction-product terminology is also used cautiously. N-A-S-H and K-A-S-H refer to sodium and potassium aluminosilicate hydrate-type gels, respectively, whereas C-(A)-S-H refers to calcium (alumino)silicate hydrate. C-(N)-A-S-H is retained where reported in the source studies, without assuming that this notation establishes a single homogeneous gel. Broad X-ray diffraction (XRD) humps and local elemental ratios from scanning electron microscopy with energy-dispersive X-ray spectroscopy (SEM–EDS) do not uniquely identify amorphous gel structure. More confident assignments require complementary evidence from methods such as Fourier-transform infrared spectroscopy (FTIR), thermal analysis, solid-state nuclear magnetic resonance (NMR), or other structural and spectroscopic techniques [40,42,50,51,52,53].

**Figure 1:**
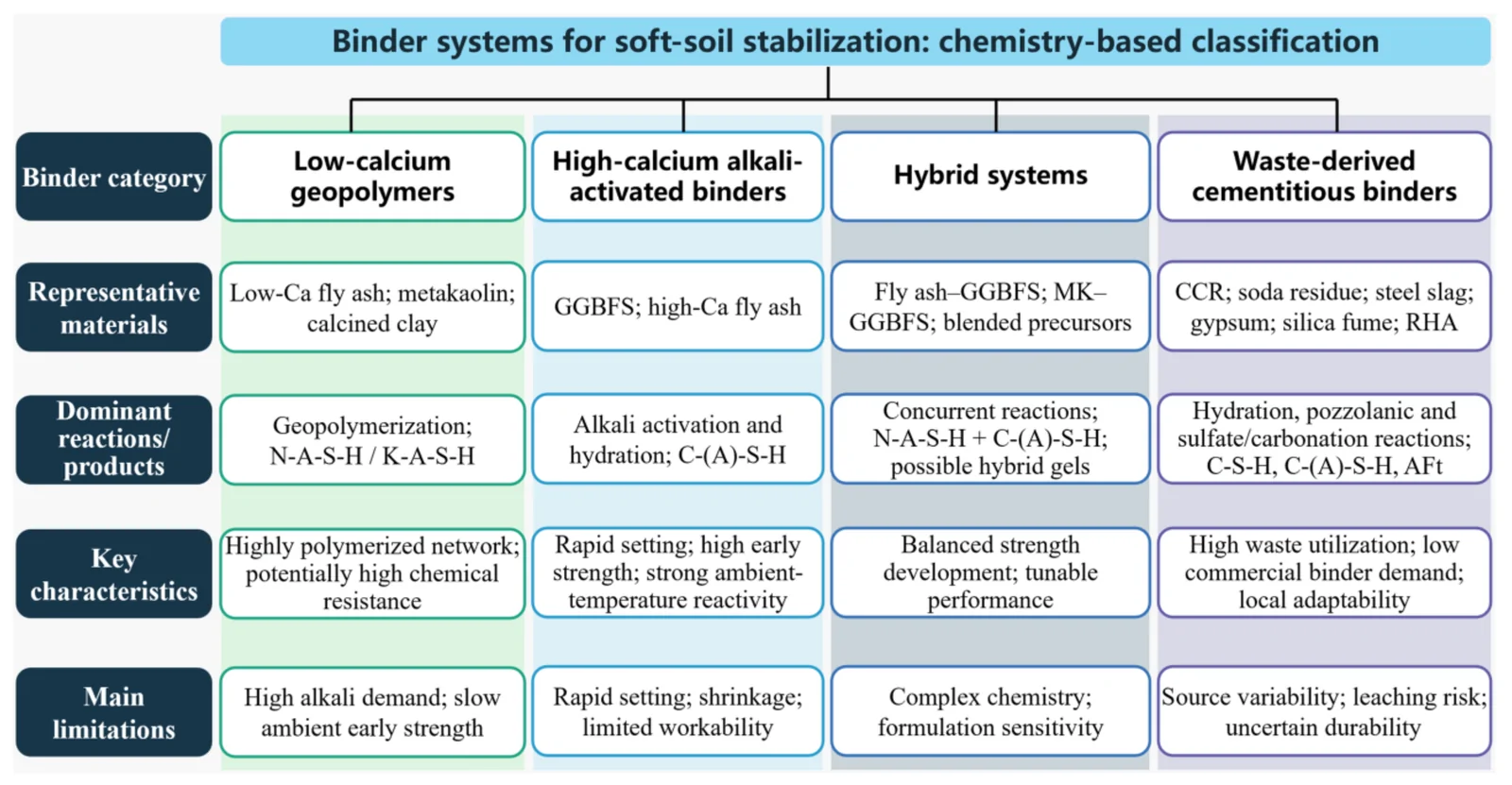
Chemistry-based classification of binder systems for soft-soil stabilization. The categories represent dominant reaction chemistry and may overlap in blended systems. GGBFS, ground-granulated blast-furnace slag; MK, metakaolin; CCR, calcium carbide residue; RHA, rice husk ash; N-A-S-H/K-A-S-H, sodium/potassium aluminosilicate hydrate; C-S-H/C-(A)-S-H, calcium silicate hydrate/calcium (alumino)silicate hydrate; AFt, ettringite.

The chemistry-based classification adopted in this review is summarized in Figure 1. It emphasizes the dominant reaction pathway while allowing overlap in blended systems.

#### 1.3.3. Evidence Selection and Synthesis

The literature base was developed through several years of topic-focused reading and searches in Scopus and Web of Science. The literature search was last updated on 20 October 2026, covering publications from 1998 to 2026. Searches were conducted iteratively using combinations of “geopolymer”, “alkali-activated”, and “soil stabilization”, without a single fixed search string. Original soil-stabilization studies were eligible if they investigated the binder systems defined in Section 1.3.2 and reported relevant reaction mechanisms, engineering performance, durability, or environmental outcomes. Studies outside this scope were excluded from the evidence map. Relevant reviews and non-soil binder studies were retained as supporting literature but excluded from the evidence counts. The evidence was organized according to soil type, precursor and activator, calcium regime, one-part or two-part preparation route, mixture variables, curing conditions, engineering performance, durability exposure, characterization method, and application scale. The qualitative appraisal considers four dimensions: (i) reporting completeness and reproducibility, including material definitions, dosage and water bases, preparation, curing, and testing; (ii) experimental robustness, including appropriate controls, replication, and reported variability; (iii) measurement and interpretation, distinguishing direct measurements from derived quantities and inferred mechanisms; and (iv) transferability across soils, exposure conditions, and application scales. Unreported information is treated as a reporting limitation, not evidence that a procedure was omitted. These dimensions inform the synthesis without a composite quality score. The evidence map and associated counts describe the original studies included in this review, encompassing core soft soils, related problematic soils, and explicitly identified soil-based materials. Fresh-state counts cover flow, bleeding, or setting measurements on soil–binder mixtures, excluding binder-only tests and compaction curves alone. Stress–strain/stiffness includes deformation curves, mechanical moduli, and shear-wave-based stiffness indicators; results derived solely from cyclic or impact loading are assigned to cyclic/dynamic. Permeability includes directly measured and consolidation-derived hydraulic conductivity. Each original publication was counted once per performance domain and application scale. A publication could contribute to multiple categories when relevant outcomes were reported; mixtures, specimens, and curing ages did not generate additional counts. The complete publication list, soil and binder descriptions, domain/scale classifications, and category totals are provided in Supplementary Table S1.

Review articles were used mainly to establish the development of the field, terminology, and broader research trends [11,12,36,41,54,55,56,57,58,59,60,61,62,63,64,65,66,67,68]. Studies on pastes, mortars, and other non-soil materials were used only where direct soft-soil evidence was limited or where they provided stronger information on reaction chemistry, material design, or sustainability.

Literature coverage was checked by tracing the reference lists of relevant reviews and key original studies and comparing the collected literature across the binder groups and performance domains considered. These checks helped identify additional relevant studies and reduce omissions. Limitations of the available literature include heterogeneous soil and test conditions, incomplete reporting, and scarce long-term field evidence. Limitations of this review include non-exhaustive coverage and potential bias in study selection and interpretation. These evidence counts describe coverage within the included literature rather than the strength or certainty of evidence.

Previous reviews have examined fly-ash-based geopolymers for clay stabilization [22], alkali-activated treatment of expansive soils [36], industrial precursors for weak soils [25], and geopolymer composites for soft-soil stabilization [23]. These reviews already discuss reaction mechanisms and engineering performance. Building on this work, the present review emphasizes the combined assessment of dominant reaction chemistry, the applicability of evidence to saturated soft clays, and performance coverage across laboratory and field scales. Its critical synthesis makes three connected contributions. First, it distinguishes binder systems according to the evidence for their dominant reaction chemistry, clarifying why waste-derived binders should not automatically be classified as geopolymers. Second, it separates direct soft-soil evidence from supporting studies on related problematic soils and non-soil materials and examines the limits of transferring their findings to saturated soft clays. Third, it links precursor composition, activation routes, and curing conditions to reaction products, soil–binder interfaces, and pore evolution, and examines how these features govern mechanical performance, durability, and environmental compatibility. Together, these contributions provide a chemistry-based framework for designing binders that meet soil-specific performance and construction requirements.

## 2. Materials, Binder Design, and Preparation

### 2.1. Soft-Soil Types and Controlling Properties

Soft soil is not an inert host for the binder. Its water content, mineralogy, organic matter, pore-water chemistry, fabric, and stress state directly affect activator concentration, precursor dissolution, ion transport, particle contact, and the development of the cemented skeleton. Among these factors, initial water content is one of the most consistently influential. In slurry-like soils, excess water dilutes the activator, increases interparticle spacing and initial pore volume, and may promote bleeding or segregation. Studies using low-calcium fly ash, fly ash–slag blends, ternary alkali-activated binders, and calcium carbide residue–fly ash systems generally report strength losses at high initial water contents [4,69,70,71]. The optimum activator and binder proportions may also shift because soil water becomes part of the reaction and mixing water. Initial water content should therefore be treated as a primary mixture-design variable rather than merely a specimen-preparation parameter.

Plasticity and mineralogy affect both water demand and soil–binder interaction. Smectite-rich and highly plastic soils have high specific surface area, strong water retention, and high cation-exchange capacity. These features can alter pore-solution chemistry, increase activator demand, and make uniform mixing more difficult. Kaolinite and illite are generally less expansive, but their contribution to reaction depends on crystallinity, structural disorder, particle size, and alkalinity. Comparative studies show that stabilization efficiency can vary markedly among natural clays, kaolin, silt, loess, and other soils even when similar binders are used [5,72,73]. In coarse soils, the strength and continuity of the binder matrix are important. In soft cohesive soils, mineral surface chemistry, adsorbed water, fabric, and soil–binder interfaces become more important.

Organic matter introduces another source of uncertainty. Humic and fulvic substances can bind reactive ions, buffer alkalinity, interfere with precursor dissolution, and weaken soil–binder interfaces. Studies on organic clay show that increasing humic acid generally reduces both cement- and geopolymer-stabilization efficiency, although slag–fly ash binders may retain better performance than cement under some conditions [5,6]. Tests on calcium carbide residue–silica fume stabilized marine organic clay further show that water content, organic content, and binder dosage interact rather than acting independently [7]. These findings support soil-specific mixture design, but they do not justify a universal organic-content limit for these binders.

Pore-water chemistry is equally important. Salinity changes ionic strength and can alter clay-particle interactions, dissolution, precipitation, and ion transport. Sulfate requires particular attention because its effect depends strongly on binder chemistry. In calcium-rich systems, excessive or delayed ettringite (AFt) formation may cause expansion and cracking. A calcium-free metakaolin geopolymer avoided detectable ettringite and substantially reduced swelling in sulfate-rich soil, illustrating the benefit of chemistry-based binder selection [9]. Sulfate can also be introduced deliberately through gypsum or sodium sulfate to promote activation or controlled AFt formation [3,28,74,75]. Sulfate is therefore not inherently beneficial or detrimental; its effect depends on Ca and Al availability, alkalinity, reaction sequence, and confinement.

Natural fabric and stress history receive much less attention than water content or index properties. Most laboratory studies use thoroughly remolded soils, which improves repeatability but alters natural bonding, anisotropy, and macrofabric. Such specimens may not reproduce either the mechanical response or the spatial variability of in situ treated soil. Studies using artificially structured clay and curing under applied stress provide evidence on the roles of bonding and stress state [31,76]. Future experiments should distinguish intrinsic material effects from specimen-state effects and report remolding history, storage, pore-fluid replacement, saturation, and curing stress more consistently.

The main soil-related constraints and their implications for binder design are summarized in Table 1. The comparison highlights the need to match binder chemistry and mixture proportions to the main constraints of each soil.

### 2.2. Precursors, Calcium Content, and Reactivity

Fly ash, GGBFS, and metakaolin are the most widely used precursors. Low-calcium fly ash is attractive because it is widely available as an industrial by-product and its spherical particles can improve mixture flow. Its reaction at ambient temperature, however, is often slow and depends strongly on the amount and composition of the reactive glassy fraction. Total SiO_2_ and Al_2_O_3_ contents are therefore poor indicators of reactivity when considered alone. Amorphous content, reactive silica, particle fineness, and the accessibility of Si- and Al-bearing phases are more informative [19,78,79]. In soil stabilization, adequate early strength in low-calcium fly ash systems often depends on activator chemistry, curing temperature, and the use of calcium-rich co-precursors [69,80,81].

GGBFS contains a highly reactive calcium aluminosilicate glass and generally reacts more rapidly than low-calcium fly ash under alkaline conditions. Dissolution of the slag and precipitation of C-(A)-S-H and related calcium-rich products can provide substantial early strength at ambient temperature. GGBFS-containing binders have therefore shown good performance in marine and high-water-content soft clays [30,32,33,71]. High slag contents, however, can shorten setting time, reduce workability, and increase autogenous or drying shrinkage [33,45]. Rapid early reaction may also lead to a later-age strength plateau or local cracking if water and activator availability become limiting. Slag is therefore most useful as a reactive calcium-rich component whose proportion is adjusted to the required setting, strength, and volume stability rather than simply maximized.

Metakaolin provides a more uniform and well-defined aluminosilicate precursor. Under suitable alkaline activation, it can form an N-A-S-H-rich network and has been used to improve the strength, stiffness, and volume stability of treated soils, including sulfate-rich soils [9,38,76]. Its main limitations are cost, the energy required for kaolin calcination, and sensitivity to water content and activator composition. Calcination conditions are particularly important. Incomplete dehydroxylation leaves less-reactive kaolinite, whereas excessive heating can promote recrystallization and reduce the amount of reactive amorphous material [82,83]. Similar considerations apply to calcined clay and thermally activated coal gangue [84,85,86]. These precursor treatments differ from post-curing sintering of geopolymer products [87].

Blended precursors are increasingly used to balance early reaction, workability, and later-age performance. Fly ash–GGBFS blends are the most common example. GGBFS supplies calcium and promotes early hardening, whereas fly ash can moderate rapid setting and contribute to continued aluminosilicate reaction. Studies of one-part fly ash–GGBFS systems indicate early formation of calcium-rich products together with continued development of aluminosilicate gels during curing [31,35,88]. Ternary and other blends introduce materials such as gypsum (including desulfurization gypsum), coal gangue, steel slag, or volcanic ash to modify sulfate availability, reactive Si and Al, calcium supply, or particle packing [1,3,28,89,90]. Such blends can perform well, but increasing the number of constituents also makes the reaction mechanism and mixture design more difficult to interpret. Claims of “synergy” should therefore be supported by appropriate single- and binary-component controls rather than inferred only from the high strength of a multicomponent mixture.

Alternative precursors can broaden the range of locally available materials. Coal gangue is rich in Si- and Al-bearing minerals but often requires mechanical, thermal, or chemical activation because kaolinite, quartz, and other crystalline phases are relatively stable [86,89,91,92]. Mine tailings and bauxite residue (red mud) have been used after alkaline, thermal, or hydrothermal treatment and in blends containing slag or silica fume [93,94,95,96,97]. Waste glass can supply reactive silica and can also be converted into a waste-derived soluble or solid silicate activator [43,98,99]. Natural volcanic ash and zeolite provide further regional options [15,100,101]. These materials are not interchangeable. Their suitability depends on mineral and glass content, particle size, soluble salts, potentially harmful elements, pretreatment requirements, and local availability.

Calcium content is useful for organizing binder systems, but it is not sufficient for predicting performance. The effect of calcium depends on its source, phase assemblage, dissolution rate, and the alkalinity and silicate concentration of the pore solution. Amorphous GGBFS can dissolve rapidly and form calcium-rich hydrates under moderate alkalinity, whereas less-reactive crystalline Ca-bearing phases may contribute more slowly [40,102,103,104,105,106]. Excessive calcium may also shorten the working time, consume soluble silicate rapidly, alter aluminosilicate polymerization, or promote precipitation of separate Ca-rich phases. Binder design should therefore consider calcium together with Si/Al, Na/Al, activator modulus, water content, and, where possible, the reactive rather than total oxide composition. No single Ca/Si or Si/Al ratio can be expected to define an optimum across different precursor–soil systems.

### 2.3. Activators and Activation Routes

NaOH and sodium silicate are the most widely used activators in two-part systems. Hydroxide provides the high-pH environment needed to dissolve reactive Si- and Al-bearing phases, while soluble silicate supplies additional silicate species and promotes gel formation. Their combined use often gives higher strength than either component alone, but only within a suitable composition range [80,81,107]. Insufficient activator limits precursor dissolution, whereas excessive alkali can increase free alkali, efflorescence, shrinkage, cost, and environmental impact. Activator modulus, usually expressed as the molar SiO_2_/Na_2_O ratio, is also important because it controls the balance between alkalinity and soluble silicate. Strength may therefore peak at an intermediate modulus. A high modulus can reduce effective alkalinity, whereas a very low modulus may provide insufficient soluble silicate [35,108].

Activator requirements depend strongly on precursor chemistry. Low-calcium fly ash and metakaolin generally require higher alkalinity or more soluble silicate than GGBFS. In a direct comparison of low-calcium coal-bearing metakaolin and high-calcium GGBFS for marine soft-soil stabilization, higher activator-to-precursor and sodium silicate-to-NaOH ratios benefited the metakaolin system but were less favorable for the GGBFS system [33]. This illustrates why nominal NaOH molarity, activator dosage, or liquid-to-solid ratio should not be compared directly across different binders. Soil water dilutes the activator, while precursor dissolution continuously changes the pore-solution composition. The effective chemical environment is therefore controlled by interactions among the soil, water, activator, and precursor, rather than by the initial activator concentration alone.

Alternative activators are increasingly studied to reduce the cost, environmental burden, and handling risks associated with concentrated NaOH and commercial sodium silicate. Sodium carbonate and sodium sulfate can activate calcium-rich precursors, although early reaction can be slower than with hydroxide activation and depends on precursor reactivity and pore-solution chemistry [109]. Calcium carbide residue (CCR), carbide slag (CS), soda residue, red mud, and other alkaline industrial wastes can supply OH^−^, Ca^2+^, Na^+^, carbonate, sulfate, or other reactive species [27,29,96,109,110,111]. Their functions, however, should be described according to the actual chemistry. CCR and CS commonly act as alkaline Ca(OH)_2_-rich sources that promote pozzolanic reactions or slag hydration. Gypsum mainly supplies sulfate and regulates reaction. Rice husk ash is normally a reactive silica source rather than an activator unless it is converted into soluble silicate.

Replacing commercial sodium silicate is particularly attractive because its production can contribute substantially to the environmental impact of alkali-activated binders. Rice husk ash and waste glass have been used as silica feedstocks for preparing soluble or solid silicate activators, with some systems achieving mechanical performance comparable to that obtained with commercial products [43,98,112,113]. Such routes should nevertheless be assessed over the complete processing chain. NaOH consumption, heating, washing, grinding, and transport can offset part of the environmental benefit. The use of a waste feedstock therefore does not by itself guarantee a low-impact activator.

One-part activation provides another route toward more field-compatible stabilization. Instead of transporting a precursor and a concentrated liquid activator separately, the precursor is premixed with a solid activator such as sodium metasilicate, solid sodium silicate, alkali-fused glass, carbonate or sulfate salts, or a preactivated waste powder [34,35,41,88,114]. Water is then added during mixing or supplied partly by the wet soil. This approach reduces the handling of bulk alkaline liquids and allows construction procedures closer to conventional dry-powder stabilization. Its performance, however, depends on dissolution of the solid activator. High soil water content may improve dissolution but dilute the pore solution, whereas low or uneven water content can cause incomplete or spatially non-uniform activation. Moisture uptake, carbonation and caking during storage, alkaline dust, and rapid setting in Ca-rich formulations also require control. Thus, one-part describes the preparation route rather than the binder chemistry and should not be interpreted as water-free or inherently safe.

Acid-activated phosphate geopolymers represent a chemically different class of inorganic binders. They can form Al–O–P- and Si–O–P–O–Al-containing networks and achieve high strength [115,116,117,118]. However, direct evidence for their use in soft-soil stabilization is very limited. Their reaction chemistry, high acid demand, and handling requirements also differ substantially from those of alkaline systems. They are therefore treated here as peripheral evidence rather than a major route for current geotechnical stabilization.

The main precursor–activator combinations discussed in this review are compared in Table 2. The comparison links activation route and reaction chemistry to practical suitability, using the distinctions established in Section 1.3.2.

### 2.4. Mixture Design, Modifiers, and Preparation

Binder content is a primary mixture-design variable, but its effect is rarely linear. Increasing binder dosage generally increases the amount and continuity of reaction products, fills large pores, and improves strength. Beyond a certain range, however, further addition may provide little benefit because available water or activator becomes insufficient, unreacted particles accumulate, shrinkage increases, or the mixture becomes difficult to mix uniformly [30,35,70,80]. Reported binder contents are also difficult to compare unless the calculation basis is stated clearly, such as dry soil mass, wet soil mass, or total solids. Whether the binder is added to or replaces part of the soil should also be specified.

Activator concentration, dosage, modulus, activator-to-precursor ratio, and liquid-to-solid ratio are closely coupled. Their effects on reaction also depend on the water already present in the soil. A NaOH concentration or activator ratio optimized for one initial water content may therefore not remain optimal after dilution by pore water. Phetchuay et al. [70] showed that increasing the water content of marine clay shifted the preferred liquid/fly ash and sodium silicate/NaOH ratios. Sukmak et al. [81] likewise found that the optimum liquid/fly ash ratio changed with the fly ash/clay ratio and used an empirical heating index combining temperature, duration, and specimen mass to describe the curing effect. These results are more useful as evidence of coupled mixture effects than as universal optimum values. Mixture design should therefore account for the complete balance of soil water, added water, activator, and reactive solids.

Chemical ratios such as Si/Al, Na/Al, and Ca/Si provide useful mechanistic guidance, but ratios based only on total oxide composition can be misleading. Quartz, mullite, crystalline kaolinite, and other phases may contribute little to reaction, so the reactive fractions of Si, Al, and Ca are more relevant than their bulk contents. In non-soil binders, NMR measurements reveal changes in Q^4^(mAl) distributions with Si/Al, while accompanying mechanical tests show that strength does not necessarily increase monotonically with Si/Al [42]. In stabilized soil, the apparent optimum is further affected by water content, adsorption at clay surfaces, and possible contributions from soil-derived Si and Al. Composition ratios should therefore be treated as design descriptors rather than fixed target values.

Modifiers can be introduced to reduce brittleness or shrinkage. Fibers can improve post-peak resistance and crack control through bridging, pull-out, and interfacial bonding. Excessive fiber content can cause agglomeration, poor dispersion, and additional voids [126,127]. Fine mineral and nanoscale additives may also act as fillers or nucleation sites and modify pore structure. For example, nano-lithium montmorillonite reduced shrinkage in a coal gangue–slag geopolymer mortar [128]. Such findings indicate possible routes for improving geopolymer-stabilized soil, but direct evidence for nanoscale modifiers in soft soils remains limited. Their dispersion, cost, and environmental burden also need to be considered before practical use.

Mixture optimization has gradually moved beyond one-factor-at-a-time testing toward orthogonal design, Taguchi methods, response-surface analysis, and machine-learning approaches [112,129,130]. These methods can identify interactions and local optima more efficiently, but their predictions remain dependent on the soil, precursor source, variable ranges, and experimental protocol used for calibration. A response surface or data-driven model developed for one material system should therefore not be treated as a transferable design equation. More useful optimization should consider several objectives simultaneously, including workability, setting time, strength, shrinkage, durability, environmental impact, and cost, rather than maximizing 7- or 28-day unconfined compressive strength (UCS) alone.

Preparation and curing also influence the measured performance. Mixing sequence determines whether the activator first contacts the precursor or is diluted by soil water. Yaghoubi et al. [71] reported higher strength when dry precursors and liquid activator were added separately to the soil rather than premixed before incorporation. Adequate mixing improves dispersion and reaction, but prolonged mixing can become detrimental once rapid reaction, heat generation, or moisture loss begins. Specimen size can also affect heat transfer and reaction kinetics [81]. Elevated-temperature curing accelerates dissolution and early strength development, particularly in low-calcium fly ash and metakaolin systems, but it is usually unsuitable for in situ ground improvement. Ambient-temperature, submerged, and stress-controlled tests can help reproduce relevant aspects of field curing. Curing stress itself can alter pore structure, stiffness, and strength [31].

Specimen conditioning and test conditions should also be reported because they affect the measured response. Saturation method, soaking or drying, confining pressure, drainage condition, and moisture state can all influence strength, stiffness, permeability, and durability. Many UCS specimens are tested under unsaturated or partly saturated conditions, whereas deep-mixed soil may remain submerged throughout curing and service. Results obtained under different conditioning and testing procedures should therefore not be compared quantitatively without considering these differences.

**Figure 2:**
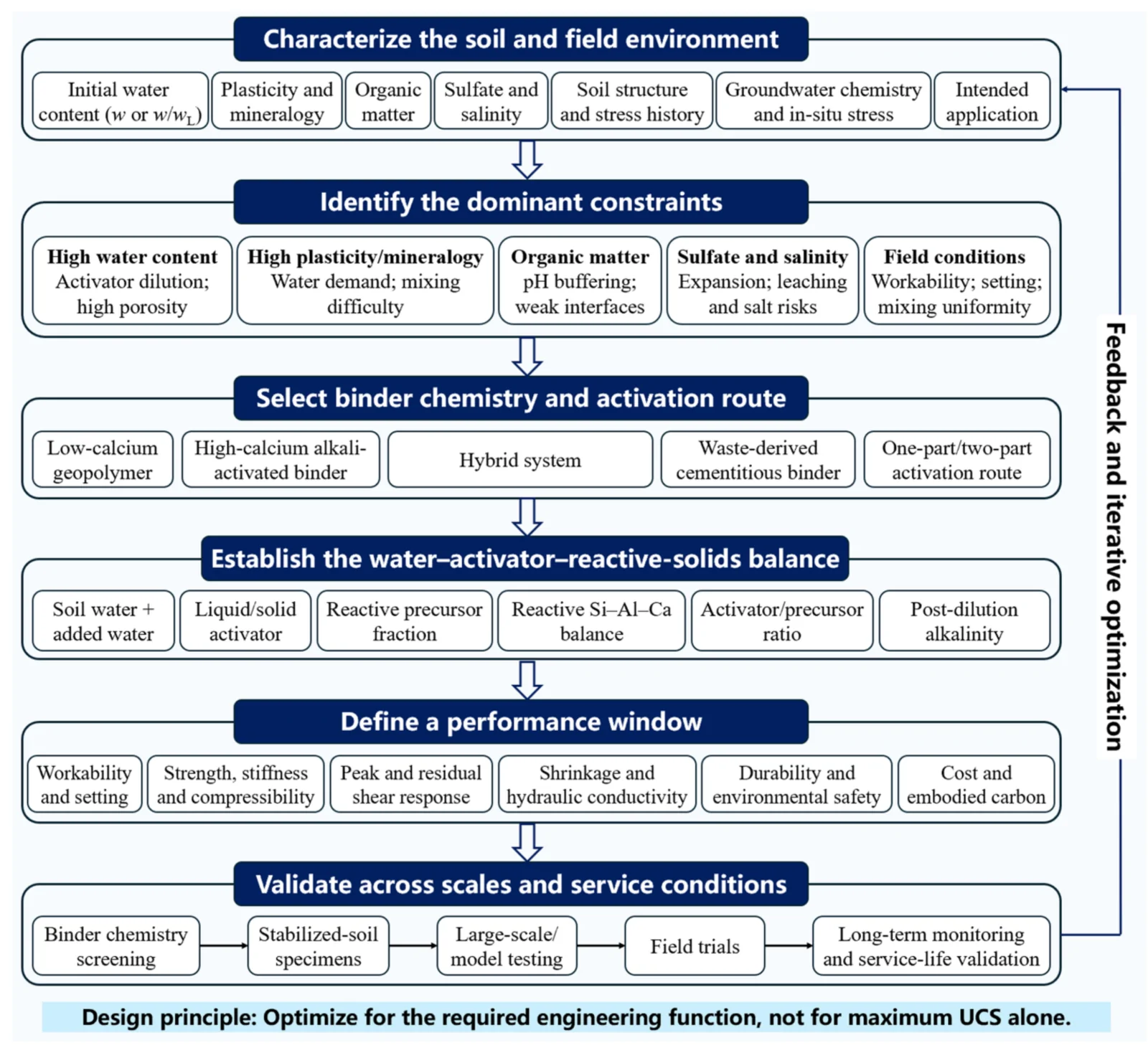
Soil-specific and performance-based framework for binder selection and mixture design. *w*, initial soil water content; *w*_L_, liquid limit; UCS, unconfined compressive strength. Binder categories follow the overlapping chemistry-based classification in Figure 1; one-part/two-part denotes the preparation route. Arrows indicate the design sequence and iterative feedback.

Taken together, these findings show that mixture design should start from the properties and water state of the soil, then select the binder chemistry and activation route, and finally optimize processing and performance for the intended application. Figure 2 summarizes this soil-specific, performance-based approach, including validation and iterative adjustment across application scales.

## 3. Engineering Performance and Governing Factors

### 3.1. Fresh-State Behavior, Setting, and Compaction

Fresh-state properties are essential for the practical use of these binders in soil stabilization. They determine whether the binder can be mixed and distributed uniformly before hardening. Important parameters include fluidity, viscosity, bleeding, setting time, working time, compactability, and mixing resistance. These properties depend on precursor type and fineness, activator chemistry, water content, soil plasticity, and temperature [35,131,132]. A formulation that develops high strength after curing may therefore still be unsuitable for field construction if it cannot be mixed, pumped, or compacted effectively.

Precursor and activator chemistry strongly influence workability and setting. Fly ash can improve flow because of its spherical particles, whereas fine metakaolin and highly reactive GGBFS can increase water demand or shorten the working time. Sodium silicate can increase viscosity and promote early structure formation. Increasing water content can improve fluidity and delay setting, but excessive water dilutes the activator, increases segregation risk, and reduces subsequent strength [35]. In GGBFS-rich systems, rapid dissolution and precipitation of C-(A)-S-H may shorten the working time to a level that is difficult to accommodate during pumping or deep mixing. Low-calcium fly ash systems can show the opposite problem, with slow reaction and insufficient early structure at ambient temperature [132]. Practical mixture design must therefore balance workability, setting, and strength rather than maximize any one property.

Compaction behavior is more relevant to stabilized subgrades and engineered fills than to slurry-like soft ground. Binder addition can change both optimum moisture content and maximum dry density, but the trends vary among studies [36,37,73,133]. Fine reactive solids may increase water demand, while replacement of soil by lower-density ash can reduce dry density. At the same time, particle rearrangement and pore filling may increase density under some formulations. These differences depend on binder density, particle aggregation, water content, and compaction effort. For high-water-content soft clay treated in situ, conventional Proctor parameters are less informative than rheology, pumpability, mixing resistance, and the ability to form a continuous and reasonably uniform treated mass. Compacted subgrades and mixed soft-soil foundations should therefore be evaluated using different fresh-state criteria.

A common limitation in mixture optimization is the selection of an “optimum” formulation from UCS alone. Min et al. [35] showed that a one-part mixture giving high compressive strength did not necessarily provide suitable fluidity or setting characteristics. The acceptable range of solid sodium silicate and water content changed when these construction requirements were considered. Fresh-state behavior and hardened performance should therefore be evaluated together. For field-oriented stabilization, the selected mixture should provide adequate workability and processing time while meeting strength and durability requirements.

### 3.2. Compressive Strength, Stiffness, and Failure Behavior

UCS is the most widely reported engineering property of soft soils treated with the binders reviewed here. Across marine clay, silty clay, dredged soil, organic clay, and kaolin-based model soils, these binders can increase UCS from the low strength of untreated soil to several hundred kilopascals or several megapascals, depending on the formulation and curing conditions [4,30,69,80,134]. The large spread in reported values reflects differences in initial water content, binder dosage, precursor reactivity, activator chemistry, curing, specimen density, and testing procedure. Absolute UCS values should therefore not be compared across studies without considering these conditions.

Strength development differs among binder systems. Low-calcium fly ash binders generally show slower early strength development but can continue gaining strength as dissolution and N-A-S-H formation proceed [19,69,80]. GGBFS-rich systems react more rapidly because calcium-rich products form at early ages, but strength may approach a plateau with continued curing [30,32,33]. Hybrid fly ash–GGBFS binders can combine rapid early hardening with continued later-age reaction, although excessive GGBFS or alkali may increase shrinkage, microcracking, or later-age strength loss [31,35,88]. Metakaolin-based systems can also develop substantial strength when adequately activated, but their performance in soil remains sensitive to water content and activator composition [9,38,76]. The strength advantage of calcium-rich precursors can therefore change with curing time. One comparison of Class C and Class F fly ashes found higher short-term strength for the high-calcium ash, whereas the low-calcium system achieved better long-term strength development [119]. For Coode Island Silt at an initial water content of 50.4%, a precursor content of 20% by dry soil mass, and 28-day curing at 23 ± 1 °C, UCS values were 0.090, 5.809, and 8.215 MPa for FA:GGBFS ratios of 100:0, 25:75, and 0:100, respectively [32]. The activator-solution/precursor mass ratio was 1.0, using a 70:30 blend of sodium silicate solution and 8 M NaOH solution. For marine clay at an initial water content of 60%, 20% GGBFS plus 5% carbide slag achieved approximately 2.3 times the 7-day UCS of 20% cement after curing at 20 ± 2 °C and 95 ± 3% relative humidity [123]. All dosages were relative to dry soil; the total binder contents were therefore 25% and 20%, respectively.

The effect of binder dosage is also nonlinear. At low dosage, reaction products form mainly around individual particles or small aggregates and do not create a continuous load-bearing skeleton. As binder content increases, gel bridges develop between soil particles, large pores are partly filled, and a connected cemented structure forms. Strength can then increase rapidly. Further addition eventually gives diminishing returns because activator or water becomes insufficient, unreacted particles accumulate, mixing becomes less uniform, or shrinkage and other defects offset the benefit of additional cementation [7,35,70,91]. This behavior explains why simple linear relationships between binder content and UCS are usually valid only over limited composition ranges.

Initial water content is one of the strongest controls on UCS. Higher water content increases initial pore volume and particle separation while diluting NaOH and soluble silicate. It can therefore reduce both the amount and continuity of reaction products and shift the optimum activator proportion [4,69,70,71]. This effect is especially important for slurry-like soft clay, where small differences in water content can produce large differences in strength. Pre-drying may improve laboratory performance but is generally impractical for in situ treatment and adds energy demand. A more realistic approach is to design binder and activator dosages from the total water content of the soil–binder system.

Curing time and temperature further control strength development. Elevated temperature accelerates precursor dissolution and shortens the induction period, particularly for low-calcium fly ash and metakaolin systems. Sukmak et al. [81] correlated strength development with an empirical index combining curing temperature, duration, and specimen mass. This index summarizes the tested heating conditions rather than measuring actual energy input, and externally heated curing is rarely practical for in situ ground improvement. For field applications, ambient-curing formulations based on suitable GGBFS contents, calcium sources, or solid activators are more practical [31,32,35,135]. Temperature history can nevertheless remain important over longer periods, as shown for calcium carbide residue–fly ash stabilized marine clay [136].

Strength gain is accompanied by a marked change in stress–strain behavior. Untreated or lightly treated soft clay commonly exhibits relatively low stiffness and ductile or strain-hardening behavior. As cementation develops, the initial stiffness and peak stress increase, while failure strain often decreases and a clearer post-peak reduction appears [72,80,91,92]. This transition reflects the formation and subsequent breakage of a bonded skeleton. High strength therefore does not necessarily imply favorable deformation behavior. Increased brittleness and strain localization may be important in embankments, pavements, and other applications where deformation compatibility is required. Fiber reinforcement can improve post-peak resistance and energy absorption by bridging developing cracks and mobilizing fiber–matrix bond and pull-out resistance [126,127].

Stiffness is reported using Young’s modulus, secant modulus, constrained modulus, and small-strain shear modulus, which should not be treated as equivalent. Stiffness generally increases with cementation and UCS, but the relationship varies with soil type, binder chemistry, curing, and the strain level at which stiffness is measured. Small-strain shear modulus can respond strongly to early bond formation even before large-strain strength has fully developed. Bender-element measurements also show that curing stress changes particle contacts, pore structure, and small-strain stiffness during hardening [31]. Shear-wave velocity therefore has potential for rapid quality control, but empirical relationships with UCS or stiffness at larger strains must be calibrated for the specific soil–binder system.

Comparisons with Portland cement show no consistent ranking. Some geopolymer and alkali-activated systems achieve higher strength than cement at similar nominal binder contents [30,70], whereas others provide comparable or lower early strength [73,120,137]. In Coode Island Silt at an initial water content of 50.4%, a 5% FA–15% GGBFS blend and a 20% cement mixture (all percentages relative to dry soil mass) gave 28-day UCS values of 5.8 and 3.0 MPa and secant moduli at 50% of peak stress (*E*_50_) of 371.3 and 243.2 MPa, respectively, after curing at 24 ± 1 °C [4]. The FA–GGBFS mixture additionally contained NaOH–sodium silicate activator solution at an activator/precursor mass ratio of 1.0, so total water contents and initial void ratios were not matched. Relative performance should therefore be assessed preferentially through within-study comparisons that explicitly account for dosage basis, total water, curing, testing age, and loading conditions. Alternative binders and Portland cement should therefore be compared at equivalent engineering performance and under comparable service conditions rather than by nominal binder percentage or short-term UCS alone.

### 3.3. Shear Strength and Triaxial Response

Evidence on shear behavior is much less extensive than that on UCS, although it is more directly relevant to bearing capacity, slope stability, and other geotechnical design problems. Direct shear and triaxial tests generally show that treatment with these binders increases peak shear resistance, mainly through the development of cementation and apparent cohesion [4,72,80,92]. Changes in friction angle are less consistent and depend on soil type, binder content, curing, and stress level. At low confinement, bonded contacts make a large contribution to strength. As confinement increases, friction, particle rearrangement, and progressive bond breakage become more important. The shear-strength improvement should therefore not be represented simply as simultaneous increases in both cohesion and friction angle.

Triaxial tests also reveal changes in deformation and failure mode. Untreated or lightly stabilized soft clay commonly shows strain-hardening or weakly peaked behavior, whereas sufficiently cemented soil tends to develop a distinct peak followed by strain softening [72,80,91,92]. Increasing binder content generally raises peak deviator stress and stiffness, but can also increase the difference between peak and post-peak resistance. Post-peak strength can also increase, although often less than peak strength. A high UCS or peak triaxial strength may therefore coexist with pronounced post-peak softening, which is important for slopes, embankments, and other systems where deformation compatibility and residual resistance matter.

Confinement and drainage conditions further influence the measured response. Jia and Chu [80] reported substantial increases in triaxial strength and marked changes in stress–strain response for fly ash-geopolymer-stabilized Tianjin clay over a range of confining pressures. Yaghoubi et al. [4] investigated both drained and undrained behavior of FA–GGBFS-stabilized high-water-content marine clay and reported improved shear strength and stiffness relative to cement-treated soil under the tested conditions. Direct-shear tests using volcanic-ash geopolymer likewise showed that binder content and curing conditions increased interparticle bonding, while normal stress increased frictional and interlocking contributions [15]. These results demonstrate that shear behavior cannot be inferred reliably from UCS alone.

Effective-stress behavior remains much less understood. During undrained loading, stabilized clay can still generate substantial excess pore pressure, while cementation, reduced compressibility, and bond degradation may modify both the pore-pressure response and the effective stress path. Few studies provide Skempton’s B-values, effective-stress strength parameters, dilation or contraction behavior, or complete stress paths in sufficient detail for critical-state or constitutive interpretation [4,80]. This lack of information is particularly important because a treatment that substantially increases total-stress strength does not necessarily produce the same change in effective-stress parameters.

The common practice of fitting a linear Mohr–Coulomb envelope from only a few stress levels also requires caution. Cemented soils can exhibit nonlinear strength envelopes, tensile resistance, pressure-dependent bond degradation, and different failure modes as confinement increases. Apparent cohesion and friction angle obtained from a limited stress range are therefore empirical parameters rather than unique material constants. Peak, post-peak, and residual strength should be distinguished, and the selected stress range should represent the intended field application. Future studies should combine drained and undrained triaxial tests, appropriate consolidation paths, pore-pressure measurements, and repeated or cyclic loading. Such data are needed to determine whether soft clay stabilized with these binders can be represented reliably using existing cemented-soil or structured-clay constitutive frameworks.

### 3.4. Compressibility, Volume Stability, and Hydraulic Behavior

Strength improvement alone is not sufficient for soft-ground treatment; deformation must also be controlled. Oedometer and consolidation tests generally show that treatment with these binders reduces compressibility and increases constrained modulus by forming a bonded skeleton and limiting the collapse of large pores [122,135,138,139]. The compression curve may develop a more distinct yield point as cementation increases. Before yielding, the bonded structure restrains deformation; once the applied stress exceeds the structural resistance, bond breakage and pore collapse can produce a marked increase in compressibility. Binder dosage, curing condition, initial void ratio, and stress level therefore need to be considered together rather than inferring settlement performance from UCS. For the same 28-day FA–GGBFS and cement mixtures discussed in Section 3.2, the reported compression indices (*C*_c_) were 0.1259 and 0.1427, respectively, from oedometer tests with loading up to 6400 kPa [4].

Curing stress is particularly relevant to field conditions. Deep-mixed soil cures under overburden and lateral confinement, whereas most laboratory specimens are cured without applied stress. Min et al. [31] showed that vertical curing stress affected drainage and settlement during curing, as well as porosity, shear-wave velocity, and strength of soft clay stabilized with one-part alkali-activated binders. Applied stress can increase particle contact and promote a denser structure, but it can also induce early consolidation, expel pore water, and redistribute the activator. Its effect is therefore not necessarily beneficial in all cases. Laboratory studies intended to represent deep mixing should include realistic curing stress, drainage, and submergence conditions.

Volume stability involves swelling, drying shrinkage, and deformation caused by changes in moisture or chemical environment. Fly ash, especially when combined with a small amount of lime, substantially reduced the swell potential of calcareous expansive soil but increased its hydraulic conductivity in related work [140]. Metakaolin-based geopolymers have reduced swelling in expansive and sulfate-rich soils [9,38,141]. Calcium-free formulations are particularly attractive for sulfate-rich soils because they limit the calcium supplied by the binder for ettringite formation [9]. Shrinkage presents a different concern. Alkali-activated slag pastes can undergo substantial drying shrinkage, depending on activator concentration and solid-to-liquid ratio [45]. Soil particles may restrain bulk shrinkage, but differential deformation between the binder and soil can generate interfacial microcracks. Shrinkage and wetting-induced deformation should therefore be measured directly in stabilized soil rather than inferred from binder-paste tests.

Hydraulic conductivity generally decreases when reaction products fill large pores and reduce pore connectivity, but the trend is not universal. Using saturated falling-head tests, Chen et al. [135] reported decreasing hydraulic conductivity with increasing binder content and curing age; at 28 days, soil treated with 20% alkali-activated grout by dry soil mass had a conductivity more than 700 times lower than untreated soil. Yaghoubi et al. [4] derived conductivity from consolidation tests and found higher values for FA–GGBFS-treated soil than for cement-treated soil, associated with its larger initial void ratio. The difference diminished as effective vertical stress increased. These findings concern different comparisons and are not necessarily contradictory. Continued reaction during moist curing can narrow or block connected pore throats, whereas aggregation may preserve interaggregate flow paths and drying-induced microcracks may bypass a denser matrix. Increasing compressive effective stress can close pores and cracks, while shear dilation or differential deformation may create preferential flow paths. Stronger particle bonding can therefore increase strength without necessarily reducing hydraulic conductivity. Additional factors include total mixture water (including activator-solution water), initial void ratio, binder composition and dosage, and curing age and conditions. These influence the initial pore volume and subsequent reaction-product formation. Soil mineralogy, permeant chemistry, saturation, and testing procedures also affect comparability, although their individual contributions remain insufficiently quantified. Consolidation rate also depends on compressibility, so faster consolidation should not be attributed to hydraulic conductivity alone. Moreover, the desirable hydraulic behavior depends on the application: seepage barriers require low and stable permeability, whereas faster drainage may be beneficial where accelerated consolidation is the objective.

Hydraulic evidence remains largely confined to laboratory testing. Most studies use small specimens after relatively short curing periods and measure hydraulic conductivity under a narrow stress range. Stress-dependent permeability, anisotropy, cracking, chemical gradients, and repeated wetting are rarely investigated. Water-retention and suction behavior are also poorly documented. These gaps are important because moisture transport affects both mechanical response and long-term chemical stability. Future studies should therefore couple compressibility, hydraulic conductivity, and volume-change measurements under field-relevant stress and environmental conditions rather than treating them as separate material properties.

### 3.5. Tensile, Bearing, Resilient, and Cyclic Performance

Tensile and flexural behavior has received far less attention than compressive strength. Available studies indicate that geopolymer and related binders can increase tensile or flexural resistance by forming a bonded matrix, but the resulting material may also become more brittle as cementation increases [28,30,126]. Fibers can partly offset this tendency by bridging cracks, redistributing tensile stress, and increasing post-cracking resistance [126]. However, splitting-tensile strength, flexural strength, tensile-to-compressive strength ratio, fracture energy, and post-cracking behavior are rarely reported together. This limits quantitative assessment of crack initiation and propagation, particularly where stabilized soil is subjected to bending or differential deformation.

For pavement and subgrade applications, bearing capacity and resilient response can be more informative than achieving a very high UCS. Studies on geopolymer, calcium carbide residue, steel slag, rice husk ash, and other alternative binders generally report increased California bearing ratio (CBR), reduced swelling, and, where measured, improved resilient modulus [112,133,142,143,144]. Some treated soils satisfy pavement or subgrade criteria after soaking, although they do not always outperform cement-treated soil [112,144]. Much of this evidence, however, comes from expansive, high-plasticity, or compacted subgrade soils rather than very soft saturated clay. Compaction effort, target density, soaking procedure, water content, and local specification also differ among studies. Reported CBR and resilient-modulus values should therefore be interpreted within their specific construction context rather than transferred directly to deep-mixed soft ground.

Direct evidence under repeated or dynamic loading remains limited. Dynamic triaxial tests on soft clay stabilized with one-part alkali-activated binders showed that dynamic shear modulus and damping ratio are affected by FA/GGBFS ratio, curing time and stress, confining pressure, cyclic-stress frequency, and cyclic-stress ratio [145]. These results confirm that cyclic response cannot be inferred from monotonic UCS alone. Bonded matrices may provide high initial stiffness but can progressively lose stiffness as cyclic strain damages particle–gel contacts. Fiber reinforcement offers a possible means of delaying crack development and maintaining residual resistance [126]. Impact tests on alkali-activated granite residual soil also indicate benefits from fiber reinforcement [127], but these findings do not establish fatigue or long-term cyclic performance in soft clays.

Environmental cycling should also be distinguished from mechanical cycling. Wetting–drying and freeze–thaw tests provide useful information on durability, but they do not reproduce the stress paths, frequencies, or accumulated strains generated by traffic or repeated foundation loading. For pavement-oriented applications, future studies should combine moisture or temperature conditioning with repeated-load, resilient-modulus, or cyclic triaxial testing and monitor the evolution of stiffness, damping, permanent strain, and damage.

**Figure 3:**
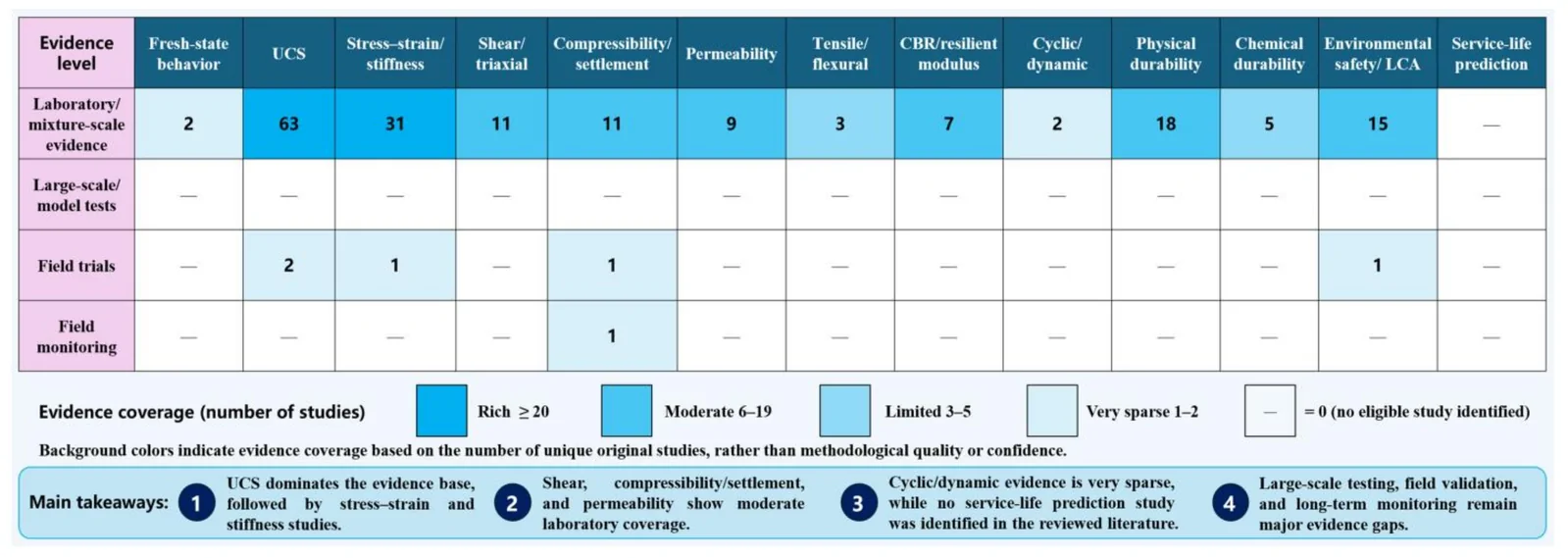
Evidence coverage across performance domains and application scales within the soil-stabilization literature included in this review. Each original publication is counted once per performance domain and application scale and may contribute to multiple cells. Reviews and non-soil studies are excluded. Physical durability includes water stability/soaking, wetting–drying, and freeze–thaw; chemical durability includes acid, sulfate, and other aggressive chemical exposures; environmental safety/LCA includes leaching, environmental-compatibility assessments, and life-cycle/carbon analyses. UCS, unconfined compressive strength; CBR, California bearing ratio; LCA, life-cycle assessment. Colors indicate rich (≥20 studies), moderate (6–19), limited (3–5), or very sparse (1–2) coverage; “—” denotes no eligible study identified. These categories describe coverage rather than methodological quality or confidence. Study-level classifications and counting rules are provided in Supplementary Table S1.

Overall, the evidence base remains strongly concentrated on UCS and short-term laboratory characterization, while tensile, resilient, cyclic, and field-scale performance are much less developed. This imbalance across engineering properties and application scales is summarized in Figure 3 and should be considered when judging the readiness of these binders for different applications. The counts indicate the coverage of eligible original studies, not methodological quality or confidence.

For example, Chen et al. [135] report defined preparation and curing conditions and triplicate UCS and permeability tests, supporting procedural reproducibility; replicate numbers alone, however, do not quantify uncertainty without dispersion estimates. In Yaghoubi et al. [4], differences in total water and initial void ratio limit attribution of performance differences solely to binder chemistry. Hydraulic conductivity was measured directly by Chen et al. [135] but derived from consolidation by Yaghoubi et al. [4], further limiting direct comparison. Similarly, model verification using the same soil and formulation at an additional water content [136] supports within-system prediction but does not establish transferability to other soils or precursor sources.

### 3.6. Governing Factors and Performance Prediction

The available evidence identifies interacting controls at the soil, binder, and curing levels. At the soil level, initial water content, mineralogy, organic matter, salinity, particle-size distribution, and initial structure determine the physical and chemical environment in which stabilization occurs. At the binder level, precursor reactivity, calcium availability, activator chemistry, binder dosage, and the balance of reactive Si and Al govern reaction and product formation. Curing and loading conditions—including time, temperature, humidity, submergence, stress, and drainage—then control the development and subsequent response of the stabilized structure [5,31,33,69,71,80].

These variables are strongly coupled. Increasing NaOH concentration can promote precursor dissolution but may also increase shrinkage, free alkali, cost, and environmental burden. Increasing GGBFS content can improve early strength while shortening working time. Additional water improves mixing and solid-activator dissolution but simultaneously dilutes the pore solution and increases initial porosity. Calcium promotes rapid formation of calcium-rich products, yet excessive calcium can alter aluminosilicate polymerization and increase sensitivity to sulfate-rich environments. Organic matter may require changes in alkalinity or binder dosage, but simply adding more alkali does not necessarily overcome its adverse effects. Single-factor relationships therefore apply only within the soil, mixture, and test conditions under which they were established.

The frequently reported rise-then-fall response with binder or activator dosage illustrates this point. An apparent optimum may correspond to sufficient precursor dissolution, formation of a continuous bonded network, improved particle packing, or a balance between beneficial reaction products and defects such as unreacted particles, shrinkage, and cracking. Similar mathematical trends can therefore arise from different physical or chemical mechanisms. Without supporting evidence from reaction progress, pore structure, or residual phases, an empirical optimum should not be assigned a unique mechanistic interpretation.

Empirical equations have been developed to relate UCS to binder dosage, water content, curing time, temperature, porosity, pH, and other variables [80,124,129,136,146]. Such models provide quantitative relationships within their calibration ranges, including strength–age correlations [32] and models incorporating water content, temperature, and curing age [136]. These relationships support local performance prediction but do not establish transferable optimum binder proportions. Across different soils and precursor sources, the reviewed evidence supports qualitative, condition-dependent design guidance rather than a validated general equation for optimum proportions. Machine-learning approaches, including artificial neural networks, random forests, and optimization-assisted models, have also reported high accuracy within the datasets used for model development [124,129]. Their performance on new soils and binder sources remains uncertain, particularly when datasets combine different soil classifications, dosage definitions, curing protocols, testing methods, and binder chemistries. Random train–test splitting can further overstate predictive capability when closely related data from the same experimental program occur in both subsets. External validation across independent soils and precursor sources is therefore more important than a high goodness-of-fit within a compiled dataset.

Data-driven methods are most useful when combined with physically meaningful descriptors and explicit limits on extrapolation. A baseline dataset for developing transferable models should include initial soil water content and liquid limit, clay fraction and mineralogy, organic matter, soil pH, and soluble salts; precursor proportions, fineness, and chemical composition, with reactivity indicators where available; and binder and activator dosages on explicit mass bases, activator composition and concentration, and total water accounting for soil water, activator solutions, and other additions. Specimen density, mixing procedure, curing age, temperature, moisture and stress conditions, and the loading and drainage conditions associated with each measured response should also be recorded. Prediction targets should also extend beyond UCS to workability, stiffness and deformation, shrinkage, durability, hydraulic response, environmental impact, and cost. Reporting prediction uncertainty and the applicable range of soils and formulations is essential if these models are to support engineering design rather than only interpolate laboratory data.

The engineering evidence reviewed in Section 3.1, Section 3.2, Section 3.3, Section 3.4 and Section 3.5 is synthesized in Table 3. It summarizes the main controls, contrasting findings, and uneven evidence coverage that limit the transfer of laboratory optima to field design.

## 4. Reaction Chemistry and Multiscale Stabilization Mechanisms

### 4.1. Reaction Pathways and Binder Distinctions

Low-calcium geopolymerization is commonly described as a sequence involving alkaline dissolution of reactive aluminosilicate phases, transport and reorganization of dissolved species, formation of Si–Al oligomers, gelation, and progressive polycondensation [17,19,50,147]. These processes overlap rather than occurring as discrete stages. The relative rates of Si and Al release depend on precursor structure, alkalinity, temperature, and solution composition [148,149,150]. Early reaction products can therefore differ in composition from the later gel. Spectroscopic and molecular-scale studies indicate that transient aluminosilicate species, soluble silicate availability, hydroxide concentration, and Si/Al ratio influence setting and network development [50,147].

In stabilized soft soil, this idealized reaction pathway is modified by the soil environment. Soil water dilutes the activator, while clay surfaces can adsorb ions and reaction species. Dissolved Si, Al, Na, and other ions must migrate through a heterogeneous pore network containing mineral surfaces, salts, organic matter, and, in many systems, calcium-bearing phases. Reaction products may precipitate around precursor particles, coat soil aggregates, fill pores, or bridge neighboring particles. The reaction is therefore spatially non-uniform, and high bulk Si and Al contents do not necessarily produce an effective load-bearing network. Sufficient precursor dissolution must be accompanied by transport and precipitation at locations that connect the soil–binder skeleton.

Calcium-rich systems follow related dissolution–precipitation processes but differ substantially in reaction products and kinetics. GGBFS and high-calcium fly ash can release Ca together with Si and Al under alkaline conditions, often favoring the formation of C-(A)-S-H and other calcium-rich products. In fly ash–GGBFS and other hybrid systems, N-A-S-H- and C-(A)-S-H-type products may coexist, evolve sequentially, or become intermixed at the nanoscale [53,88]. Calcium can also act as a charge-balancing cation without detectable C-S-H formation, as reported in blended fly ash geopolymers [40]. These reactions are appropriately described as alkali activation, but not all such systems represent classical low-calcium geopolymerization. The distinction becomes even more important for waste-derived binders in which hydration and pozzolanic reactions make a major contribution to hardening.

Calcium carbide residue-based systems illustrate this difference. CCR supplies Ca(OH)_2_, which provides alkalinity and reacts with reactive silica and alumina from fly ash, silica fume, plant ash, or other sources. The resulting binders may contain C-S-H, C-(A)-S-H, calcium aluminate hydrates (C-A-H), carbonate phases, and, depending on sulfate availability, sulfate-bearing products [7,27,130,151]. Their strength can therefore arise predominantly from pozzolanic and hydration reactions rather than from formation of an alkali-aluminosilicate network. Similar caution is required for systems containing soda residue, steel slag, gypsum, or other Ca- and salt-rich wastes. These binders may perform well, but their reaction mechanism should be identified from the actual reactants and products rather than inferred from the generic label “geopolymer.”

These overlapping reaction pathways can thus be compared in terms of their principal reactants and products. Portland cement hydration starts from clinker minerals and forms C-S-H, portlandite, AFt, and related hydrates. Pozzolanic reactions consume Ca(OH)_2_ together with reactive Si and Al to form secondary calcium-bearing hydrates. Low-calcium geopolymerization forms charge-balanced alkali-aluminosilicate networks without requiring calcium, whereas alkali-activated GGBFS is dominated by calcium-rich hydrate formation from a glassy precursor dissolved under alkaline conditions. In blended soil binders, these pathways can occur simultaneously and interact with one another. The absence of a separately added alkaline activator does not imply a non-alkaline environment, because waste constituents such as CCR can supply alkalinity [123]. Conversely, alkalinity alone does not establish geopolymerization. Distinguishing hydration from pozzolanic contributions also requires consideration of the reacting precursor phases and their evolution during curing; C-(A)-S-H formation or Ca(OH)_2_ consumption alone is not diagnostic.

**Figure 4:**
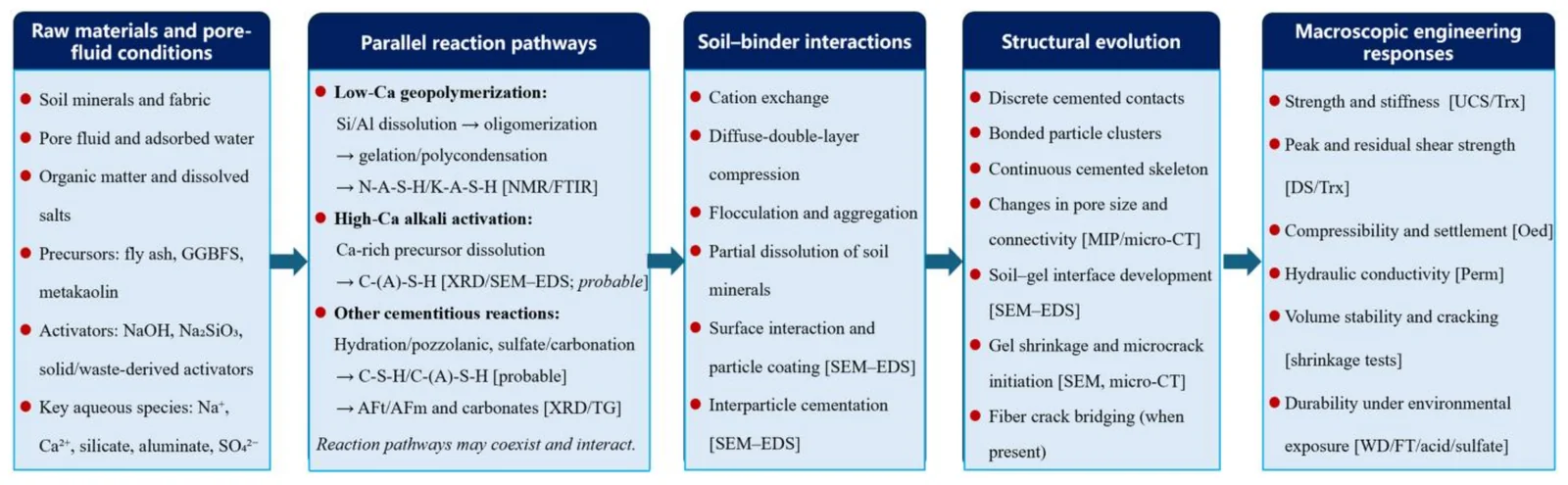
Parallel reaction pathways and multiscale stabilization mechanisms in soft soils treated with geopolymer, alkali-activated, and waste-derived binders. Reaction pathways may coexist. Bracketed labels identify representative characterization methods or engineering tests and do not themselves establish unique gel identities. Amorphous gel assignments based mainly on XRD and SEM–EDS are considered probable rather than definitive. GGBFS, ground-granulated blast-furnace slag; N-A-S-H/K-A-S-H, sodium/potassium aluminosilicate hydrate; C-S-H/C-(A)-S-H, calcium silicate hydrate/calcium (alumino)silicate hydrate; AFt, ettringite; AFm, layered calcium aluminate hydrate phases. XRD, X-ray diffraction; SEM–EDS, scanning electron microscopy with energy-dispersive X-ray spectroscopy; FTIR, Fourier-transform infrared spectroscopy; TG, thermogravimetry; NMR, nuclear magnetic resonance; MIP, mercury intrusion porosimetry; micro-CT, X-ray microcomputed tomography. UCS, unconfined compressive strength; Trx, triaxial testing; DS, direct shear; Oed, oedometer testing; Perm, permeability testing; WD, wetting–drying; FT, freeze–thaw.

Accordingly, the reaction framework adopted in this review treats the major binder chemistries as parallel and potentially interacting pathways rather than a single universal geopolymerization process, as summarized in Figure 4. This distinction provides the chemical basis for linking reaction products to soil–binder interaction, pore-structure evolution, and macroscopic engineering performance in the following sections.

### 4.2. Reaction Products in Low-Calcium, High-Calcium, and Hybrid Systems

N-A-S-H is generally the principal binding gel in sodium-activated low-calcium fly ash and metakaolin geopolymers. It consists of a disordered aluminosilicate network of SiO_4_ and AlO_4_ tetrahedra, with alkali cations providing charge balance for tetrahedral Al. Its composition and network structure depend on precursor reactivity, Si/Al and Na/Al ratios, water content, and curing conditions. Solid-state ^29^Si and ^27^Al magic-angle-spinning (MAS) NMR shows that changes in Si/Al alter the distribution of Q^4^(mAl) environments. Maximum strength does not necessarily coincide with the highest nominal Si/Al ratio [42]. In stabilized soil, N-A-S-H can bind particles and aggregates, fill pores, and form bridges across particle contacts, although its development may be slow at ambient temperature when low-calcium precursors are poorly reactive.

C-(A)-S-H is the dominant binding product in alkali-activated GGBFS and many other high-calcium systems. Compared with C-S-H in neat Portland cement, slag-derived C-(A)-S-H can contain more Al and have a lower Ca/Si ratio [51]. Its composition also depends on precursor and activator chemistry. Relatively rapid precipitation of this gel contributes to early setting and strength development. High GGBFS contents can produce a dense cemented matrix, but shrinkage may also promote microcracking. Drying shrinkage has been documented in alkali-activated slag pastes [45]. Depending on precursor composition, Mg-bearing phases such as hydrotalcite-like products and sulfate-bearing phases such as AFt or AFm may coexist with the main calcium silicate hydrate gel.

Hybrid systems, including fly ash–GGBFS blends, contain more complex reaction products. N-A-S-H- and C-(A)-S-H-type gels may form simultaneously or sequentially, and Ca, Na, Si, and Al can occur within closely intermixed reaction regions [31,32,88,92]. Terms such as C-(N)-A-S-H are therefore useful descriptive shorthand, but they should not automatically be interpreted as proof of a single homogeneous hybrid gel. Conventional SEM–EDS samples a finite interaction volume, so an elemental composition containing Ca, Na, Si, and Al may represent adjacent or intermixed phases rather than one distinct product. More definitive identification requires complementary evidence from solid-state NMR, high-resolution microscopy, or other structural methods. From an engineering perspective, the important point is that calcium-rich products generally support rapid early skeleton formation, while continued aluminosilicate reaction can contribute to later-age development.

Crystalline reaction products can also influence performance. AFt may fill pores and contribute to early strength in sulfate- or gypsum-containing systems [3,28,75,111], but excessive or delayed formation can cause expansion. Carbonate phases such as calcite may result from carbonation of Ca-rich constituents and can either densify the structure locally or indicate consumption of reactive alkaline or calcium-bearing phases. Zeolitic or other crystalline aluminosilicate phases may develop during prolonged or hydrothermal curing of some low-calcium systems. Their formation is not inherently beneficial, because crystallization can change the amount and structure of the amorphous binding phase. Residual quartz, mullite, kaolinite, slag, fly ash, and other unreacted particles also remain common and may act as inert fillers, partially reactive reservoirs, or local weak zones depending on their size and distribution.

Reaction-product identification therefore requires caution. A broad amorphous hump in XRD cannot by itself distinguish N-A-S-H, C-(A)-S-H, or mixed gels, particularly in soil where crystalline minerals contribute strong diffraction peaks and background signals. FTIR band shifts can indicate changes in Si–O–T (T = Si or Al) bonding but are not unique fingerprints of a particular gel. Thermogravimetry and derivative thermogravimetry (TG–DTG) can provide evidence for bound water, portlandite, carbonates, and some hydrate decomposition ranges, although overlapping reactions complicate interpretation. SEM–EDS is valuable for examining morphology and local elemental composition but does not uniquely resolve amorphous gel structure. Mechanistic assignments should therefore rely on converging evidence from complementary techniques rather than on a single characterization method. Direct structural evidence from soil–binder systems remains limited. In kaolin stabilized with alkali-activated high-calcium fly ash, Coudert et al. [121] combined NMR and complementary analyses to support calcium-associated silicate chains. NMR also detected new tetrahedral Al environments, which the authors suggested may be incorporated into these chains. Prasanphan et al. [42] provided NMR evidence for aluminosilicate networks in non-soil binders, which does not directly establish their formation in stabilized soils. Accordingly, N-A-S-H, C-(A)-S-H, and hybrid-gel assignments based mainly on XRD and SEM–EDS are treated as inferred assignments rather than definitive identifications.

For future studies, priority should be given to combined ^29^Si and ^27^Al MAS NMR to assess silicate connectivity and Al coordination, using untreated-soil and precursor spectra and comparisons across curing ages to help distinguish newly formed products from residual materials. FTIR and TG–DTG provide complementary evidence for bonding changes and hydrate evolution, but cannot independently establish gel identity. NMR assignments likewise require corroboration where signals overlap.

### 4.3. Soil–Binder Interactions and Skeleton Formation

Before substantial cementitious products form, the alkaline pore solution can alter clay-particle interactions. Changes in pH affect clay-edge charge, while exchange of Na^+^, K^+^, or Ca^2+^ at clay surfaces alters diffuse-double-layer behavior. Ca^2+^ is particularly effective in promoting flocculation and aggregation, while increased ionic strength generally reduces electrostatic repulsion between particles. These early physicochemical changes can modify fabric, rheology, and stiffness before longer-term cementation becomes dominant [27,130,146,152]. Their importance depends on clay mineralogy, pore-water chemistry, and the type and concentration of activator.

The extent to which soil minerals participate chemically in binder formation is strongly system-dependent. In some high-calcium fly ash–kaolin mixtures, kaolin behaved mainly as a relatively inert component [121]. Under sufficiently alkaline conditions, however, reactive or disordered clay surfaces can release Si and Al that may contribute to subsequent reaction products [5,37,127,153]. Such participation depends on mineral type, crystallinity, particle size, alkalinity, exposure time, and solution composition. Bulk mineralogy alone therefore cannot establish that the soil itself contributes substantially to geopolymerization or other binding reactions.

Organic matter can interfere with stabilization at several stages. Humic substances may coat mineral and precursor surfaces, bind calcium and other reactive ions, and buffer pore-solution alkalinity. Organic-rich regions may also remain as relatively weak or compliant inclusions after curing. These effects can reduce reaction efficiency, interrupt binder continuity, increase porosity, and lower strength [5,6,7]. Although some slag–fly ash systems retain better strength than cement at comparable organic contents, their performance can still decline as organic interference increases.

At the mesoscale, stabilization is governed by the development of a connected composite skeleton. Reaction products coat soil and precursor particles, bridge neighboring particles or aggregates, and progressively fill larger voids. At low binder contents, cemented contacts remain isolated and the original soil skeleton still governs deformation and failure. As binder dosage and curing time increase, bonded clusters can become interconnected and form a continuous load-bearing network. At still higher levels of cementation, the response becomes increasingly matrix-controlled, and failure may localize through the hardened binder or along soil–binder interfaces [76,80,91,92]. This structural transition helps explain the common change from ductile or strain-hardening behavior toward higher stiffness, clearer peak strength, and post-peak softening.

Chemical bonding, particle rearrangement, and pore evolution therefore act together rather than independently. Fine precursor particles can fill larger voids before reaction begins, while flocculation may initially create larger aggregates and interaggregate pores. Continued precipitation then fills, bridges, or subdivides these voids. Curing stress can further change particle contacts, pore volume, and the evolving bonded structure [31]. The final skeleton is consequently governed by the coupled effects of reaction chemistry, particle packing, consolidation, and moisture redistribution. This multiscale interaction provides the link between reaction products and the pore-structure changes discussed in the following section.

### 4.4. Microstructural Evolution and Mechanism–Performance Relationships

The engineering response of stabilized soil develops through the combined evolution of reaction products, particle contacts, pores, and cracks. During early curing, precursor dissolution and initial precipitation modify particle surfaces and contacts. Mercury intrusion porosimetry (MIP) studies commonly indicate pore refinement, reflected in a shift toward smaller accessible pore-entry sizes as binder dosage or curing time increases [35,92,135]. Such refinement is often accompanied by higher strength and stiffness. Total porosity alone, however, does not determine performance; pore connectivity, tortuosity, interface quality, and crack distribution can be equally important.

SEM provides direct visual evidence of particle coating, bridging, aggregation, and cracking, but its interpretation requires caution. The observed area is small, and drying, impregnation, polishing, or fracture preparation can alter weak soil–binder structures. MIP mainly reflects accessible pore-entry sizes rather than the full sizes of pore bodies, and high intrusion pressures may disturb weak structures. N_2_ adsorption probes a different and generally finer range of accessible pores. X-ray computed tomography (CT) can provide three-dimensional information on larger pores and cracks but cannot resolve nanoscale gel structure. More reliable microstructural interpretation therefore comes from combining complementary chemical, spectroscopic, imaging, and pore-characterization methods rather than relying on a single technique [35,71,92,135].

Crack evolution is particularly important because densification and damage can occur simultaneously. Rapid reaction, autogenous or drying shrinkage, moisture gradients, and mechanical loading can generate microcracks even in a matrix with low measured porosity. GGBFS-rich and highly activated binders may develop a dense structure but can also be susceptible to shrinkage [45]. Subsequent wetting–drying or chemical exposure may enlarge existing defects or weaken soil–binder interfaces. Fiber reinforcement can bridge and deflect cracks [126,127]. Fine or nanoscale modifiers may also improve pore structure and shrinkage behavior, as reported for related geopolymer mortars [128]. Apparent crack filling during later curing or immersion should not, however, be interpreted automatically as self-healing unless recovery of mechanical or transport properties is demonstrated.

The link between microstructure and macroscopic performance is therefore not one-to-one. Pore refinement and improved bond continuity generally increase strength and stiffness and can reduce hydraulic conductivity, but an overly stiff or shrinkage-prone matrix may become vulnerable to cracking. Calcium-rich products often accelerate early strength development, yet their long-term stability depends on the chemical environment. Likewise, a highly polymerized aluminosilicate gel does not guarantee high soil strength if reaction products remain concentrated around precursor particles rather than forming effective bridges between soil aggregates. Mechanistic interpretation should therefore consider reaction chemistry, pore structure, interfaces, and damage together and relate them directly to measured engineering behavior over time.

Table 4 summarizes the information provided by these techniques, their complementary roles, and the limits of the mechanistic conclusions they support.

## 5. Durability, Environmental Safety, and Sustainability

### 5.1. Water Stability and Wetting–Drying Durability

Water stability is critical because many stabilized soft soils remain saturated or are exposed to repeated changes in moisture. Water exposure can reduce strength through leaching of soluble alkalis and weakly bound species, clay swelling, softening of interfaces, and crack development during subsequent drying. Continued reaction during immersion or cycling may partly offset these effects when unreacted precursor remains available. The measured response therefore reflects competition between ongoing reaction and moisture-induced deterioration rather than a simple loss of strength with exposure [16,108,154].

Available studies show that resistance to water depends strongly on binder chemistry and dosage. For fly ash–GGBFS-stabilized soft soil, Wu et al. [108] reported a water-stability coefficient of 87.53% at a binder content of 25%. The optimized mixture retained a UCS of approximately 1.6 MPa after eight wetting–drying cycles. These values demonstrate good performance under the specific test conditions, but they should not be treated as general durability thresholds. Under cycles involving 5 h of immersion and 42 h of drying at 70 °C, the 28-day-cured CCR-only mixture disintegrated during the third cycle. The CCR–fly ash mixture initially gained strength but declined after five cycles, whereas its alkali-activated counterpart continued gaining strength through seven cycles [16]. This contrast shows that the durability of a waste-derived binder cannot be inferred from its individual constituents or initial UCS alone.

Similar dependence on formulation and exposure conditions has been observed in marine waste silt stabilized with CCR and plant ash. Increasing binder dosage improved water resistance, whereas organic matter and ambient temperature substantially affected resistance to wetting–drying cycles [154]. These results also highlight the importance of the soil itself: high water content, organic matter, mineralogy, and pore structure can alter both reaction progress and damage accumulation. Maheepala et al. [113] assessed wetting–drying resistance and heavy-metal leaching in stabilized expansive soil. Such related-soil evidence should not be transferred quantitatively to saturated soft clay because moisture transport and cracking conditions differ.

Comparison among studies is further complicated by inconsistent testing procedures. Drying temperature and relative humidity, wetting medium, degree of saturation, cycle duration, number of cycles, specimen dimensions, and failure criteria vary widely. Oven drying can introduce thermal and shrinkage damage that is more severe than many field moisture cycles, whereas simple immersion does not reproduce hydraulic gradients or fluctuating groundwater conditions. Durability assessment should therefore report the exposure protocol together with strength retention, mass and dimensional changes, visible cracking or disintegration, and, where possible, changes in pore structure and hydraulic behavior. Strength-retention ratios should identify the reference age and testing moisture condition. Unexposed controls of the same age can help distinguish exposure effects from continued hardening. Exposure histories should reflect the intended application.

### 5.2. Freeze–Thaw and Chemical Durability

Evidence for freeze–thaw durability is much less developed than that for strength and is concentrated mainly on compacted expansive, saline, and subgrade soils rather than saturated deep-mixed soft clay. Available studies indicate that geopolymer and alkali-activated treatment can improve freeze–thaw resistance by strengthening particle contacts and reducing the connectivity of larger pores [100,133]. This benefit is nevertheless sensitive to saturation, pore structure, binder dosage, and soil type. A dense matrix may still suffer frost damage when high saturation limits pressure relief or water migration supports ice growth. Results obtained from compacted or partly saturated soils should therefore not be transferred directly to saturated soft-ground applications.

Acid exposure presents a different deterioration mechanism. In calcium-rich systems, acid attack can decalcify C-(A)-S-H and related hydrates, dissolve carbonate phases, and progressively increase porosity. Low-calcium aluminosilicate gels are less susceptible to decalcification but can still undergo dealumination, ion exchange, and alkali loss. Tests on one-part GGBFS–fly ash-stabilized soft clay exposed to HNO_3_ and H_2_SO_4_ for up to 240 days showed that a GGBFS/FA ratio of 80:20 provided the best acid resistance among the investigated mixtures [155]. The advantage was attributed to a favorable balance between reaction products and pore structure rather than simply to lower calcium content. Under severe H_2_SO_4_ exposure at pH 2, however, even the better-performing mixtures eventually disintegrated [155]. Acid resistance therefore depends on exposure severity and duration as well as binder composition.

Sulfate resistance likewise depends on binder chemistry, pore structure, and the availability of Ca and Al. Under external sulfate exposure, cement-stabilized soil can deteriorate through formation of expansive sulfate-bearing phases, cracking, and surface damage. In one comparative study, alkali-activated slag–fly ash-stabilized soil showed better resistance than cement-treated soil under Na_2_SO_4_ exposure. The 9:1 GGBFS/FA mixture retained 86.6% of its pre-exposure UCS after 90 days in 2.5% Na_2_SO_4_ solution [8]. Calcium-free metakaolin geopolymer provides a different strategy: in sulfate-rich clay, it substantially reduced swelling and no ettringite was detected, unlike conventional calcium-based stabilization [9]. These results support the use of low-calcium or carefully proportioned binders where sulfate-induced expansion is a concern, but they do not imply that all such binder systems are sulfate-resistant.

Sulfate can also be introduced deliberately as part of the binder system. Gypsum, sulfate salts, and sulfate-bearing wastes can promote early formation of AFt or modify activation and thereby improve early strength in selected formulations [3,28,75]. The distinction between beneficial and damaging sulfate reactions is therefore important. Early and spatially distributed formation within available pore space may contribute to hardening, whereas continued or delayed formation after a rigid skeleton has developed can generate expansive stress. Sulfate durability should consequently be assessed for the specific Ca–Al–sulfate balance and exposure condition rather than inferred from the presence or absence of sulfate alone.

Evidence for chloride and mixed marine exposure is even more limited. Chloride can alter pore-solution chemistry and interact with calcium- and aluminum-bearing phases, while soluble salts may also affect leaching and moisture transport. One study of geopolymer-improved clay assessed resistance after 28 days of immersion in 5% NaCl solution and reported retained mechanical performance, but such short-term immersion provides only preliminary evidence [77]. Coastal ground may be exposed to chloride and sulfate together with wetting–drying, groundwater flow, and sustained or cyclic stress. Long-term studies that combine these actions are therefore needed to assess durability under marine and coastal field conditions.

### 5.3. Long-Term Aging and Dimensional Stability

Long-term performance remains weakly documented because many soil-stabilization studies terminate at 28, 56, or 90 days. Curing studies extending to 240 days for CCR–fly ash-stabilized marine soft clay [136] and 365 days for alkali-activated fly ash-stabilized soil [120] demonstrate prolonged hardening, but not resistance to sustained loading or environmental deterioration. The exposure tests summarized in Section 5.1 and Section 5.2 provide condition-specific durability evidence. For example, acid-exposure tests extended to 240 days, but even the better-performing mixtures disintegrated in H_2_SO_4_ at pH 2 [155]. Expected durability benefits from gel chemistry or pore refinement remain subject to validation. Laboratory exposure durations and cycle counts cannot be translated directly into service life without field calibration. Neither these tests nor the limited field observations discussed in Section 6.4 establish multi-year stability under coupled mechanical and environmental actions.

Strength development at later ages depends on binder chemistry and exposure history. Slowly reacting fly ash-rich systems may continue gaining strength as precursor dissolution and gel formation proceed, whereas highly reactive GGBFS-rich systems often develop most of their strength earlier. Later performance may also be affected by moisture redistribution, shrinkage-induced cracking, leaching, carbonation, and changes in reaction products. A high 28-day UCS therefore does not guarantee stable long-term performance. Long-term assessment should track strength together with stiffness, volume change, cracking, and hydraulic behavior.

Dimensional stability is a particular concern but has received much less attention than strength. Alkali-activated GGBFS pastes can exhibit substantial drying shrinkage, and higher activator concentration was associated with greater shrinkage in the investigated system [45]. Although soil particles can restrain bulk deformation, this restraint may transfer shrinkage into local tensile stresses and cracking at soil–binder interfaces. Evidence from non-soil geopolymer mortar shows that nano-lithium montmorillonite can markedly reduce drying shrinkage in coal gangue–GGBFS systems [128], suggesting a possible mitigation route. However, such results should not be transferred directly to stabilized soft soil without verification because water content, confinement, soil fabric, and shrinkage restraint differ.

Other aging processes are even less understood. Efflorescence reflects the migration of soluble salts with pore water and their precipitation at exposed surfaces, while carbonation can alter alkalinity and convert calcium-bearing phases to carbonates. Depending on where these processes occur, they may densify local regions or contribute to chemical gradients and cracking. Creep and stress relaxation are particularly important omissions because untreated soft soil is strongly time-dependent and stabilized ground remains subjected to sustained stress throughout its service life. Long-term studies should therefore examine strength, creep, shrinkage, carbonation, alkali migration, hydraulic conductivity, and microstructural changes under realistic stress, moisture, temperature, and CO_2_ conditions.

### 5.4. Leaching and Environmental Compatibility

The use of industrial by-products and waste-derived activators introduces environmental concerns that are not captured by mechanical performance alone. Potential issues include the release of heavy metals, soluble salts, residual alkalis, and other constituents from either the precursor or the activator. Stabilization can reduce contaminant mobility through physical encapsulation, adsorption, precipitation, and incorporation into reaction products. In 64-day monolithic tank tests with renewed deionized water, Tang et al. [16] reported lower Cu, Cr, and As release from CCR–fly ash than from its alkali-activated counterpart, whereas Pb release was lower in the activated system. Cumulative metal release remained below the non-hazardous monolithic-waste limits used in that study; these waste-acceptance criteria do not establish groundwater compatibility. Related non-soil waste-derived binders have also shown contaminant-immobilization potential, providing supporting rather than direct soil-stabilization evidence [75,156]. These results are encouraging, but they remain strongly dependent on waste source, binder chemistry, curing, and exposure conditions.

Environmental compatibility should therefore be assessed more broadly than by measuring selected heavy metals. In deionized-water batch extractions, Yaghoubi et al. [4] reported arsenic concentrations of 0.04 and <0.01 mg/L and eluate pH values of 11.8 and 12.0 for FA–GGBFS- and cement-stabilized clay, respectively. These concentrations are not directly comparable with cumulative release per unit area from monolithic tank tests. Neither this study nor Tang et al. [16] quantified Na^+^ or K^+^ release; pH alone cannot establish their concentrations or cumulative release. Leachate pH, Na^+^ and K^+^ release, chloride, sulfate, and total dissolved solids should also be assessed because they may affect groundwater, surrounding soil, and vegetation even when regulated metal concentrations are low. This issue is particularly relevant to residues such as soda residue and other saline industrial wastes, which can contain substantial amounts of chloride and soluble alkalis [29,157,158]. Their suitability depends on whether the release of these mobile constituents remains acceptable under the intended service conditions.

Leaching behavior is also time- and condition-dependent. A single batch extraction provides only a short-term measure and may not represent transport from a stabilized mass exposed to groundwater over many years. Tank-leaching and pH-dependent tests can provide information on release rates and controlling mechanisms. Cracking, carbonation, wetting–drying, acidification, and changes in groundwater chemistry may further alter contaminant mobility by exposing previously encapsulated particles or changing the stability of binding phases. Long-term environmental assessment should therefore consider both the initial chemical state and its evolution during service.

Classification of a precursor as a non-hazardous waste does not remove the need to evaluate the stabilized product. Waste composition can vary among sources and production batches, while activator dosage and local groundwater chemistry can change leaching behavior. For field application, environmental acceptance should therefore be integrated with engineering design: the selected mixture should satisfy mechanical and durability requirements while also meeting appropriate pore-water and leaching criteria under representative exposure conditions.

### 5.5. Life-Cycle Impacts, Cost, and Circularity

The environmental benefit of geopolymer and alkali-activated soil stabilization is substantial in some systems but cannot be assumed from binder type alone. Replacing Portland cement with fly ash, GGBFS, coal gangue, tailings, or other industrial by-products can reduce clinker demand, divert waste from disposal, and lower associated emissions. For soft marine clay, Phetchuay et al. [70] reported lower CO_2_ emissions for CCR–fly ash stabilization than for cement when mixtures were compared at similar strength levels. A more recent life-cycle assessment of CCR-based binders likewise showed lower impacts than conventional cement or lime for several formulations, although the results were sensitive to material processing, transport, and energy source [16]. Environmental performance is therefore determined by the complete stabilization system rather than by precursor replacement alone.

The activator can offset a substantial part of the benefit obtained from waste precursors. Commercial sodium silicate and NaOH are energy- and emission-intensive, and sodium silicate can make a major contribution to the climate-change impact of alkali-activated materials [13,14]. Activator burdens are accounted for through their quantities and corresponding production inventories. For example, Fořt et al. [13] included production inventories for commercial NaOH and sodium silicate in a non-soil composite assessment, whereas Tang et al. [16] included NaOH consumption, glass processing, and thermal treatment in the inventory for a waste-derived silicate activator. Comparisons also require consistent system boundaries and clear assumptions about NaOH production route and concentration, electricity source, transport distance, and allocation of environmental burdens to industrial by-products. GGBFS is a notable example: assigning it no upstream ironmaking burden gives a more favorable result than allocating part of that burden through economic or other allocation procedures. Ghadir et al. [15] estimated climate-change impacts of 75.11 and 78.22 kg CO_2_-eq per m^3^ of compacted clayey soil for volcanic-ash geopolymer and Portland cement stabilization, respectively, at comparable 28-day shear strength after dry curing at 45 ± 2 °C. Their common production-stage boundary included raw-material extraction, processing, and upstream transport but excluded delivery to the construction site, construction activities, and service. NaOH production alone contributed 64.97 kg CO_2_-eq per m^3^ to the geopolymer total. This strength-matched comparison does not establish equivalent durability or service life. Such results caution against describing geopolymer stabilization as inherently low-carbon.

The functional unit is therefore central to meaningful comparison. An impact expressed per kilogram or tonne of binder does not account for the amount needed to achieve the required ground performance. A more appropriate functional unit is a defined volume of treated soil meeting specified requirements for strength, settlement, hydraulic conductivity, durability, and service life [15,16]. Material and energy inputs should then be calculated for that unit. For pavement applications, treated layer thickness, resilient response, and design traffic may also need to be included. For deep mixing, binder dosage should be considered together with column geometry, mixing energy, material losses, and quality-control requirements. Performance-normalized assessment is particularly important because a nominally low-impact binder may lose its advantage if substantially higher dosage or more intensive processing is required.

Economic feasibility is equally site-specific. Locally available fly ash, GGBFS, or other wastes can reduce raw-material cost, whereas drying, grinding, calcination, chemical activation, and long-distance transport can offset these savings. Commercial NaOH and sodium silicate may account for a large fraction of binder cost. One-part systems can reduce the handling and transport of liquid activators, but they require production and storage of solid activators, including protection from moisture. Waste-derived alkali or silicate sources may reduce dependence on commercial chemicals and improve resource efficiency [16,98,109,159], but the energy and chemicals used in pretreatment must be included. Variability in waste composition can also create additional costs for characterization, mixture adjustment, and quality control.

Circularity should likewise be evaluated beyond the simple percentage of waste incorporated. Some widely used precursors, particularly high-quality GGBFS and fly ash, are regionally limited, already have established markets, or may become less available as industrial processes change. Transporting such materials over long distances or diverting them from higher-value uses can weaken the circularity argument. A more robust strategy is to prioritize locally available residues that can be converted into safe and durable stabilizers with limited processing. The sustainability value of these binders therefore lies in using local wastes to meet engineering, durability, and environmental-safety requirements with demonstrable life-cycle benefits.

## 6. Engineering Applications and Scale-Up

### 6.1. Shallow and Deep Mixing

Deep soil mixing (DSM) is one of the most direct routes for applying geopolymer and alkali-activated binders to soft-ground improvement because existing DSM equipment is already designed to mix cementitious materials with in situ soil. Laboratory studies of fly ash–GGBFS binders have reported strength and stiffness gains and explored conditions relevant to deep mixing [30,31,71]. Two-part binders are generally suited to wet mixing, where the activator and precursor can be introduced as a slurry or through separate feeds. One-part binders can be premixed before construction and supplied as powders or used to prepare a slurry. For dry delivery, field suitability depends on sufficient soil water for activator dissolution and uniform distribution of the constituents.

The main challenge is that laboratory mixture performance does not directly represent a field-mixed column. Small laboratory specimens are usually prepared with controlled water content and intensive mixing, whereas field treatment produces spatial variations in binder dosage, water content, mixing energy, and soil composition. Clay lumps, organic-rich layers, shells, sand seams, and groundwater flow can further reduce uniformity. Curing conditions also differ. Deep-mixed soil hardens under overburden and lateral confinement and may remain submerged throughout curing. Laboratory evidence shows that curing stress can significantly change pore structure, stiffness, and strength [31]. Field-scale validation should therefore focus not only on nominal binder composition but also on the actual soil–binder state produced during construction.

Construction parameters become part of the material design at this scale. Binder delivery rate, slurry concentration or powder feed, penetration and withdrawal rates, blade rotation speed, number of mixing passes, and mixing energy all affect column uniformity and strength. The usable working time is also important for highly reactive GGBFS-rich mixtures because rapid setting may interfere with pumping or mixing. Conversely, slowly reacting low-calcium systems may not provide sufficient early strength for construction. Laboratory optimization should therefore define a practical range of workability, setting time, early strength, and long-term performance before full-scale trials are undertaken. Trial columns should then be used to calibrate binder dosage and construction parameters under the actual groundwater, stress, and soil conditions.

Shallow mass stabilization provides a related route for treating dredged mud, excavated soil, reclaimed ground, and materials intended for working platforms or engineered fill. Waste-derived binders containing fly ash, GGBFS, steel slag, CCR, soda residue, and other industrial by-products have been investigated for these high-water-content or subgrade-oriented applications [2,111,158]. Many of these systems are more appropriately described as waste-derived cementitious or alkali-activated binders rather than classical geopolymers. Their scale-up challenge is nevertheless similar: local variability in water content, salinity, organic matter, and waste composition must be accommodated while maintaining adequate mixing and predictable performance. For both shallow and deep mixing, successful scale-up therefore depends on integrating binder chemistry with construction process control rather than transferring a laboratory “optimum” mixture directly to the field.

### 6.2. Subgrades, Embankments, and Soil Reuse

Road subgrades and embankments impose different requirements from deep-mixed foundations. Workability, compaction, soaked strength, volume stability, and resistance to moisture and traffic loading are usually more important than achieving very high UCS. Studies on expansive soils and clayey subgrades treated with geopolymer, alkali-activated, and other waste-derived binders report improvements in UCS, CBR, swelling control, and durability [112,133,142,143,144]. However, much of this evidence comes from compacted expansive or high-plasticity soils rather than the saturated soft clays that form the core of this review. Binder dosages, curing conditions, and compaction procedures also vary considerably. Mixtures intended for large-volume earthworks should therefore be evaluated against pavement- or embankment-specific criteria rather than selected from laboratory UCS alone.

Field evidence remains limited. Miranda et al. [160] constructed an 80 m long and 2.5 m wide full-scale (sub)base trial using five soil–binder systems, including three alkali-activated fly ash sections. The trial confirmed that these binders could be placed and compacted using field procedures and showed that in situ moisture control, curing environment, and construction sequence affected the resulting performance. The alkali-activated sections achieved mechanical responses broadly comparable with conventional binders, although the commercial activators substantially increased cost in some mixtures [160]. Because the treated material was a compacted sandy lean clay rather than very soft saturated soil, the study provides valuable scale-up evidence but should not be taken as direct validation for saturated soft-clay stabilization.

Dredged and excavated soils offer a particularly attractive reuse route because stabilization can convert a high-water-content waste into construction fill or reclamation material [1,2,3,111]. Their high water content can facilitate mixing but also dilutes alkaline activators and increases the initial pore volume. Salinity, organic matter, and particle composition may vary within the same source. Large-scale treatment therefore requires control of both material composition and water state. Depending on the initial condition and intended use, dewatering, staged binder addition, or combined mechanical and chemical treatment may be needed. The target properties should also reflect the final function, whether the material is used as low-strength fill, an embankment core, a roadbed material, or structural backfill.

Waste-derived binders may support this reuse route by combining dredged or excavated soils with industrial residues. Laboratory studies have demonstrated the feasibility of GGBFS–fly ash–desulfurization gypsum and other multi-waste systems for high-water-content soils [1,3]. Gu et al. [161] reported small-scale outdoor stabilization of dredged sludge using fluorogypsum and calcined shell powder, supplemented by 72-h seawater immersion and laboratory leaching tests. Treatment reduced the release of Cd, Cr, Cu, Pb, and fluoride under the tested conditions. These findings provide preliminary application evidence but do not establish long-term field durability or groundwater compatibility. Broader use of these waste-derived binders requires large-batch mixing, control of spatial variability, and verification of settlement, hydraulic behavior, volume stability, and leaching. These requirements are especially important where treated material is exposed to fluctuating groundwater levels or placed close to surface-water systems.

Other applications have less direct evidence for soft-soil treatment. Studies of lightweight soils, foamed concrete, and waste-derived cementitious materials provide related information on workability, hardening, durability, and waste reuse [156,162,163,164]. Their mixture proportions and preparation methods differ from those used in the soft-clay studies reviewed here. Geopolymer grout formulations have also been investigated [165], yet direct evidence for permeation or jet grouting with geopolymer binders in soft clay remains limited. Low soil permeability is a further constraint on permeation grouting in intact soft clay. Cristelo et al. [134] reported a field application of an alkali-activated fly ash binder using jet grouting. This provides direct field evidence for the tested soil–binder system, although broader validation in saturated soft clays remains limited. Low hydraulic conductivity in selected laboratory mixtures supports their potential for seepage control [1,69], but does not establish long-term hydraulic-barrier performance. Verification requires direct saturated permeability testing under service stresses and relevant permeant chemistry, including after deformation or environmental cycling, together with field-scale assessment of barrier continuity.

The literature provides much more evidence from laboratory strength tests than from full-scale construction and long-term monitoring. Table 5 summarizes the current application readiness of the main routes discussed in Section 6.1 and Section 6.2 and identifies the validation still needed before broader engineering adoption.

### 6.3. Construction Routes and Field Safety

The construction route depends on binder chemistry and how the activator can be stored, delivered, and mixed with soil at field scale. Conventional two-part systems use solutions of NaOH, sodium silicate, or both and allow relatively precise control of activator composition. Their field use, however, introduces practical constraints. Concentrated alkaline liquids require dedicated preparation, storage, pumping, and corrosion-resistant equipment, while sodium silicate can increase slurry viscosity and complicate delivery. Preparation of NaOH solution is also strongly exothermic. In addition, the water already present in soft soil dilutes the activator and must be included in mixture design. The full-scale (sub)base trial further showed that activator volume, in situ water content, transport, and construction sequence can affect implementation and cost [160].

One-part systems can simplify this process by premixing the precursor with a solid activator and using added water or water already present in the soil [34,35,88]. This reduces bulk alkaline-liquid handling at the construction site and brings preparation closer to conventional dry-binder practice. It may be particularly useful where high-water-content soil can provide part of the water needed for activator dissolution. The advantage is conditional, however. Insufficient or non-uniform moisture can cause incomplete dissolution and local under-activation, whereas excessive soil water reduces effective alkalinity. Solid activators may also absorb moisture or CO_2_ during storage, cake, and lose reactivity. Fine alkaline powders can create dust and contact hazards. One-part systems may therefore simplify handling, but their safety and field performance still require verification.

Waste-derived activators and alkaline co-reactants can reduce dependence on commercial NaOH and sodium silicate, but they introduce additional quality-control demands. CCR, soda or other alkali residues, red mud, and activators made from waste glass can vary in alkalinity, soluble-salt content, particle size, moisture condition, and reactive composition [29,98,125,157,166]. These variations can affect setting, workability, reaction rate, leaching, and final strength. Where several wastes are combined, controlled preprocessing and factory preblending may therefore be preferable to proportioning multiple variable materials directly on site.

Field control should consequently focus on a small set of measurable properties that reflect both construction quality and binder reaction. Water content, binder delivery rate, slurry or mixture density, pH, electrical conductivity, and rheological or flow properties can provide rapid information during production, but their engineering meaning must be calibrated for the specific soil–binder system. They should complement, rather than replace, strength, stiffness, hydraulic conductivity, and durability testing. The most suitable construction route is therefore the one that provides reliable binder distribution, adequate working time, manageable safety risks, and reproducible field performance under the actual soil moisture and groundwater conditions.

### 6.4. Scale Effects, Quality Control, and Monitoring

Scale-up changes both the material response and the way performance must be evaluated. Specimen-size effects observed during thermal curing of clay–fly ash geopolymer show that heat and moisture transfer can influence reaction and strength development [81]. These results should not be transferred directly to field elements, but they demonstrate that laboratory curing conditions and specimen dimensions can affect measured performance. In large treated volumes or columns, temperature, drainage, groundwater flow, and spatial variability may produce reaction conditions that differ substantially from those in small molds.

Quality control should therefore include both the construction process and the properties of the treated ground. Reliance on UCS tests of recovered cores alone is insufficient because coring samples only a small part of a heterogeneous treated mass and may underrepresent weak or poorly mixed zones. During construction, binder delivery, water content, mixing energy, penetration and withdrawal rates, and other application-specific parameters should be recorded. The rapid measurements described in Section 6.3 can support process control, while wet grab samples provide material for controlled curing and testing. After treatment, UCS and stiffness testing can be complemented by penetration tests, geophysical methods, and other integrity measurements where appropriate. Laboratory results for soft clay stabilized with one-part alkali-activated binders show that changes in shear-wave velocity accompany changes in curing stress, porosity, and strength [31]. This makes wave-based methods promising for quality assessment, but the relationship between velocity and engineering properties must be calibrated for each soil–binder system rather than treated as universal.

Field monitoring is needed to determine whether laboratory improvements are maintained under actual loading and environmental conditions. The monitoring program should reflect the intended application and may include settlement, lateral deformation, pore pressure, stiffness, groundwater or leachate chemistry, and changes in strength with time. For pavement and embankment applications, surface deformation and repeated-load response are also relevant. Field monitoring of steel slag–rice husk ash-stabilized high-plasticity subgrade soil over approximately 150 days, for example, has demonstrated the value of settlement measurements for evaluating treated embankment performance [144]. Full-scale alkali-activated (sub)base trials have likewise shown that field construction and in situ curing can produce behavior that cannot be captured fully by laboratory testing [160]. Such studies remain uncommon, particularly for saturated soft clay treated with these binders.

Economic feasibility at field scale is closely linked to constructability. As discussed in Section 5.5, material cost alone is not an adequate basis for comparison. Binder production and pretreatment, activator consumption, transport, storage, mixing energy, equipment requirements, safety measures, construction rate, quality control, and avoided disposal costs should all be considered. A mixture with excellent laboratory strength may therefore be unattractive if it requires expensive activators, has a short working time, or demands unusually intensive mixing. Field-scale optimization should seek the required performance with acceptable construction reliability and total project cost rather than maximize strength.

**Figure 5:**
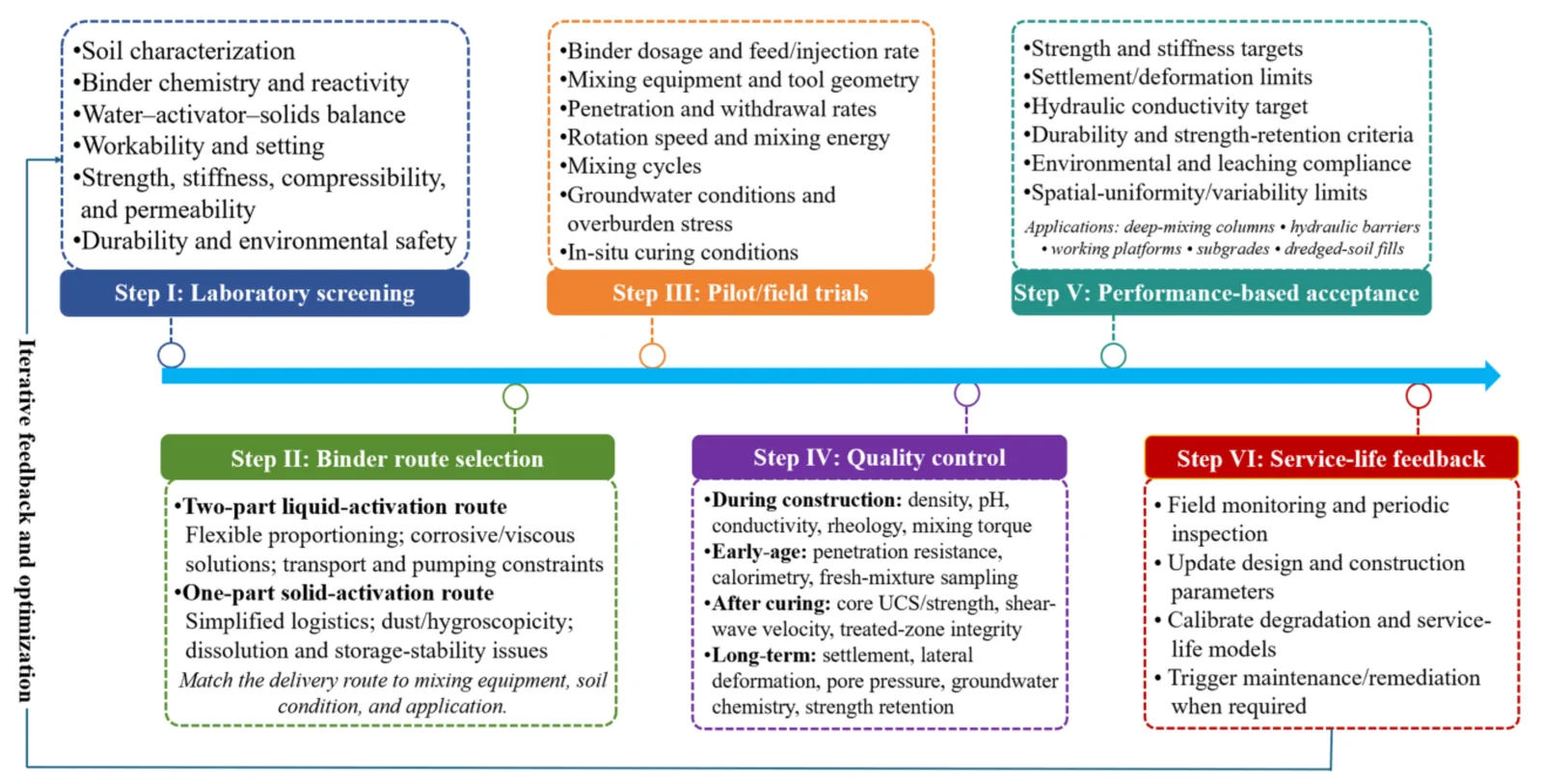
Laboratory-to-field scale-up, quality control, monitoring, and closed-loop performance validation framework for soft-soil stabilization using geopolymer, alkali-activated, and waste-derived binders. UCS, unconfined compressive strength. Colors distinguish implementation stages; the main arrow indicates progression, and the return arrow indicates iterative feedback.

The transition from laboratory formulation to engineering use is therefore an iterative process rather than a one-way transfer of an optimized mixture. Laboratory design should define an initial performance window, and field trials should identify scale-dependent deviations. Construction records and monitoring should then guide adjustments to mixture proportions and construction parameters against predefined performance requirements. This laboratory–field–feedback framework is summarized in Figure 5, which links material design, construction control, quality assessment, field monitoring, and performance validation into a closed-loop scale-up process.

## 7. Research Gaps and Future Directions

### 7.1. Terminology, Testing, and Reporting

Consistent terminology is needed for meaningful comparisons across studies. Binder classification should reflect calcium content and sources, activation chemistry, and evidence for dominant reaction products, following the distinctions in Figure 1. One-part and two-part labels describe preparation routes and should be reported separately from binder chemistry (Table 2).

Minimum reporting should include soil origin and mineralogy, initial water content relative to the liquid limit, and organic matter and salt contents. Precursor phase composition and reactivity should also be documented. Activator composition should be reported on an oxide basis, with clearly defined mass bases for binder and activator dosages. The total water balance must account for water in the soil, activator solution, and other additions. Mixing procedures, curing conditions (age, temperature, moisture, and stress), specimen density, and degree of saturation also need to be recorded.

Test protocols should distinguish compacted subgrade soils from soft clays stabilized at high water contents. Specimen preparation, pre-test conditioning, and loading and drainage conditions require clear definitions. Durability comparisons need consistent exposure procedures, including cycle number and duration, temperature, and solution chemistry and concentration. Strength-retention calculations should identify the reference strength, age, and testing moisture condition. Unexposed controls of the same age can help separate exposure effects from continued hardening. Replicate counts and variability should be reported for all tested mixtures.

### 7.2. Soil-Specific Binder Design

Binder design should account for soil properties and construction conditions, as outlined in Figure 2. High-water-content clays require control of activator dilution, while organic clays require assessment of alkali demand and reaction inhibition. Low-calcium binders may be suitable for sulfate-rich soils, subject to verification of swelling resistance and durability. Saline dredged soils require assessment of pore-water chemistry and leaching. A practical screening procedure could combine measurements of pH buffering capacity, organic matter, and soluble salts with small-scale mixing trials to guide precursor and activator selection.

Binder formulations selected from paste tests need validation in the target soil under realistic mixing and curing conditions. Optimization should balance workability, setting time, strength, shrinkage, and safe handling. Research should identify dosage ranges that meet construction and performance requirements despite variations in soil water content and precursor properties. This would provide a more useful basis for field application than a single formulation selected solely for maximum UCS.

### 7.3. Sustainable Activation Strategies

Future activation strategies should reduce reliance on commercial sodium silicate and NaOH while controlling total alkali input and handling risks. Promising options include silicates derived from waste glass or rice husk ash [43,112,113]. Other routes include carbonate or sulfate activation of suitable calcium-rich precursors [109] and the use of CCR or soda residue as sources of alkalinity and calcium [16,75]. Waste-derived solid activators also offer a route to one-part formulations [98], although storage stability and dissolution in soil water require further assessment.

Comparisons should use equivalent engineering performance and account for activator production, pretreatment energy, transport, storage, and leaching. For calcined clay and coal gangue, pretreatment should balance energy consumption with the reactivity achieved. Mechanical treatment and lower-temperature calcination deserve evaluation, including their effects on the binder dosage needed to meet performance targets. Regional waste availability, processing capacity, and transport distances should also inform material selection, since the benefits of a locally sourced formulation may not extend to other locations.

### 7.4. Durability and Service-Life Prediction

Most durability studies examine individual exposures over short periods. Future tests should assess interactions between mechanical loading, groundwater flow, and environmental exposure under conditions relevant to the intended application. Relevant exposures include wetting–drying, temperature changes, exposure to sulfate or chloride solutions, and carbonation. Exposure sequence, drainage conditions, and stress level should be defined clearly. Measurements should track stiffness, hydraulic conductivity, crack development, leaching, and residual shear strength alongside UCS.

Service-life models should link ongoing binder reactions and degradation to transport through evolving pores and cracks. They should distinguish continued hardening from deterioration and relate these processes to changes in engineering performance. Accelerated tests need validation against long-term field observations, particularly where increased exposure severity may change the dominant degradation mechanism. Creep, stress relaxation, and progressive bond damage under cyclic loading remain important gaps, especially for predicting accumulated deformation and long-term stability.

### 7.5. Multiscale Modeling and Performance-Based Design

Coupled reaction–transport models should describe activator dilution, precursor dissolution, gel precipitation, and ion migration while accounting for heat generation and pore evolution. Thermodynamic models can support predictions of calcium-rich phase assemblages, but disordered N-A-S-H gels and soil–binder interactions need better representation and validation. Molecular simulations should link gel structure to measurable properties that can inform models at larger scales.

Constitutive models should represent bond development and degradation, stress dependence, strain softening, and time-dependent deformation. Parameter calibration should combine UCS, triaxial, and oedometer tests with shear-wave measurements to constrain strength, compressibility, and small-strain stiffness. Validation requires independent tests covering relevant stress paths, drainage conditions, and curing ages. Together, these models should link binder composition and curing conditions to the engineering response of stabilized soft soils. Performance-based design can then guide mixture selection and treatment design using application-specific requirements for strength, stiffness, deformation, hydraulic conductivity, and durability.

### 7.6. Field Validation and Data-Driven Design

Further full-scale trials are needed for shallow and deep mixing, subgrade construction, and dredged-soil reuse. The framework in Figure 5 provides a basis for linking these trials to quality control and long-term monitoring. Trials should record equipment settings, mixing energy, binder distribution, soil water content, curing temperature, groundwater conditions, and spatial variability in treated-soil properties. Acceptance criteria should be defined alongside mixture design. Field measurements of deformation, material performance, and groundwater quality should then be used to assess compliance.

Open databases should retain soil properties, material sources, mixture definitions, test conditions, and uncertainty. Explainable machine learning could help examine interactions among soil and binder variables. Independent validation across soils, binder sources, and field datasets should establish predictive accuracy and limits of applicability. Physics-informed models should incorporate relevant mass and charge balances. Multi-objective optimization should combine workability, strength, shrinkage, and durability with life-cycle cost, carbon emissions, and environmental safety. The output should be a set of feasible mixtures that meet these requirements under expected material and construction variability, with prediction uncertainty taken into account.

## 8. Conclusions

**Findings supported by current evidence**

(1) The reviewed binders comprise four overlapping groups: low-calcium geopolymers, high-calcium alkali-activated binders, hybrid systems, and waste-derived cementitious binders. Their classification should reflect evidence for the dominant reaction chemistry. Waste-derived systems may harden mainly through hydration, pozzolanic reactions, sulfate reactions, or carbonation and should not automatically be classified as geopolymers. Across these systems, particle bonding, pore filling, and changes in soil fabric contribute to the development of a connected soil–binder skeleton.

(2) Direct laboratory studies of stabilized soft soils generally show increased strength and stiffness and reduced compressibility, but stronger cementation can also increase brittleness and post-peak softening. Hydraulic conductivity shows less consistent trends because flow also depends on pore connectivity, cracking, and stress. Initial water content, considered both as an absolute value and relative to the liquid limit, strongly influences activator dilution and particle spacing. Other key soil parameters are clay fraction and mineralogy, organic matter content, soil pH, and soluble salts, particularly sulfates. Their effects interact with precursor reactivity, calcium availability, and curing conditions. Fly ash–GGBFS blends can balance early strength, workability, and continued reaction at ambient temperature, but their optimum proportions remain specific to the soil and application.

Environmental and economic advantages depend on the complete treatment system, including activator production, precursor processing, transport, binder demand, construction, and service conditions. One-part systems can reduce bulk alkaline-liquid handling, while waste-derived activators can reduce demand for commercial chemicals. Their practical benefits depend on storage stability, effective dissolution, mixture uniformity, and control of material variability.

**Unresolved questions and evidence gaps**

(3) Evidence is concentrated on laboratory UCS, stress–strain behavior, and routine microstructural observations. Shear behavior, compressibility, and hydraulic conductivity have moderate laboratory coverage. Tensile and cyclic behavior, creep, coupled durability, and long-term field performance remain poorly documented. Evidence for CBR, freeze–thaw resistance, and subgrade field performance comes mainly from related problematic soils. Detailed gel-structure and binder-workability interpretations also draw on non-soil studies. Transfer of these findings to saturated soft clays therefore requires verification. Amorphous gel identities cannot be established uniquely by XRD and SEM–EDS alone.

**Recommendations for future research**

(4) For practical implementation, long-term field validation and durability under coupled mechanical and environmental conditions should take priority, because short-term laboratory gains do not establish sustained field performance. Further priorities depend on the application: creep for settlement-sensitive ground under sustained loads, cyclic response under traffic or seismic loading, and tensile/fracture behavior in crack-sensitive applications such as hydraulic barriers. Solid-state NMR can strengthen gel assignments, while pore and interface measurements are needed to connect reaction products with engineering behavior.

Mixture selection should satisfy construction, strength, deformation, and durability requirements together. Comparisons with cement should use equivalent engineering performance and consistent life-cycle boundaries, alongside assessments of leaching and groundwater compatibility. Broader adoption requires standardized terminology and reporting, validated predictive models, coupled durability testing, and full-scale trials. These efforts should support soil-specific, performance-based design that connects mixture selection with construction control and long-term monitoring.

## Figures and Tables

**Table 1:** Soft-soil characteristics, stabilization challenges, and implications for binder design.

Soil Characteristic	Primary Physicochemical/Structural Effects	Consequences for Stabilization	Binder-Design Implications	Representative Evidence	Evidence Coverage
High initial water content	Dilutes the activator and increases interparticle spacing, pore volume, and effective liquid-to-solids ratio	Generally reduces strength and may shift optimum binder/activator proportions	Account for total water; optimize binder and activator dosage for the actual soil water content rather than transferring a fixed optimum	Higher water content reduced UCS and shifted the effective binder/activator range in soft-clay systems [69,70,71]	Broad
High plasticity/ smectite-rich mineralogy	High surface area, water retention, and cation-exchange capacity increase water demand and soil–binder interaction; mineral reactivity varies with clay type	May hinder mixing and delay strength development; treatment efficiency is strongly soil-dependent	Characterize mineralogy and plasticity/activity and adjust water, activator, and binder dosage accordingly	Treatment efficiency varied substantially with clay mineralogy and plasticity [5,72]	Moderate
Organic matter	Buffers alkalinity, interferes with reaction chemistry, and weakens cemented-skeleton continuity	Generally retards reaction and reduces strength; effects depend on organic type, content, and water condition	Determine organic content and pore-fluid pH; use soil-specific mixture design rather than universal thresholds	Increasing humic acid reduced pH and UCS [6]; mineral–organic interactions and coupled water–organic effects were reported [5,7]	Moderate
Sulfate-rich soil/ pore fluid	Sulfate interacts with available Ca and Al and may form expansive sulfate-bearing phases	Expansion and cracking risk depends strongly on binder chemistry and reaction sequence	Quantify sulfate and available Ca/Al; consider low-Ca or calcium-free binders where expansion is critical; assess swelling as well as strength	Calcium-free metakaolin geopolymer markedly reduced swelling and showed no detectable ettringite [9]; sulfate resistance remained formulation-dependent [8]	Moderate
Salinity and dissolved salts	Alter ionic strength and dissolution/precipitation equilibria; soluble ions may migrate or leach	Effects on reaction and long-term performance depend on salt type, concentration, and binder chemistry	Characterize pore-water salinity and major ions; reproduce representative pore-fluid conditions and include salt-exposure or leaching tests where relevant	Direct evidence remains limited; NaCl-exposure studies mainly address post-curing durability [77], while some field-condition simulations did not include water-quality effects [71]	Limited
Natural fabric and stress history	Natural bonding, anisotropy, sensitivity, and stress state govern particle contacts and destructuration	Remolded, unstressed specimens may not reproduce field curing and stress–strain behavior	Report initial state and remolding history; use representative curing stress and complement remolded tests with structured or field-relevant validation	Artificial structure altered strain-softening and failure behavior [76], while curing stress affected drainage, settlement, UCS, and shear-wave velocity [31]	Limited

Note: Evidence coverage reflects the breadth and directness of the supporting literature rather than methodological quality or confidence. Reported numerical optima are soil- and formulation-specific. UCS = unconfined compressive strength.

**Table 2:** Qualitative comparison of representative precursor–activator systems for soft-soil stabilization.

Binder System/Representative Precursors	Activation Route	Dominant Products/ Evidence	Ambient Reactivity/ Workability	Engineering Implications	Key Limitations/Risks	Terminology/Caution
Low-calcium geopolymer—Class F FA, MK/calcined clay [9,69,119,120]	Mainly NaOH and/or Na_2_SiO_3_; calcium-free formulations possible	N-A-S-H-dominated aluminosilicate gel; gel identity in many soil studies is inferred mainly from XRD/SEM–EDS	Generally slower early reaction but sustained later-age strength development; activator viscosity may hinder mixing	Good later-age strength; calcium-free systems may be advantageous in sulfate-rich soils	Slow early strength in some FA systems; liquid-alkali handling, viscosity, cost, and shrinkage	Classical geopolymer where a low-Ca alkali-aluminosilicate network dominates
High-calcium alkali-activated binder—GGBFS, high-Ca FA, and Ca-rich slag blends [33,121,122,123]	NaOH/Na_2_SiO_3_ solutions or Ca(OH)_2_-rich activators such as CS	Ca-rich silicate/aluminosilicate hydrates, commonly C-(A)-S-H/C-S-H; C-A-H and sulfate-bearing phases may also occur	Rapid ambient reaction and high early strength; working-time window may be narrow	Strong early-age performance and good ambient-curing compatibility; some solid Ca-rich activators are suitable for dry-mixing concepts	Rapid setting, workability loss, and shrinkage; excessive alkali or Ca may impair later performance	Prefer alkali-activated slag/high-Ca binder rather than assuming an N-A-S-H-type geopolymer
Hybrid FA–GGBFS system—low-Ca FA + GGBFS [4,30,32,108]	Typically NaOH/Na_2_SiO_3_ solution	N-A-S-H and C-(A)-S-H-type products may coexist; mixed Ca–Na aluminosilicate gels are possible	GGBFS accelerates early reaction, while FA can moderate setting and support continued reaction	Useful balance of early strength, later-age development, and water stability; highly relevant to soft-soil mixing	Sensitive to FA/GGBFS ratio, water content, and activator chemistry; excessive GGBFS or alkali may cause rapid setting or shrinkage	Hybrid system; a single hybrid gel should not be assigned from XRD/SEM–EDS alone
One-part solid-activated route—FA, GGBFS, or their blends [31,34,35,88]	Solid NaOH, sodium metasilicate, or sodium silicate mixed with the precursor; water added during use.	Products depend on precursor chemistry and may be N-A-S-H-rich, C-(A)-S-H-rich, or mixed	Avoids bulk liquid activator but depends strongly on available water and dissolution of the solid activator	Reduces liquid-alkali transport and handling and is promising for field-oriented mixing	Alkaline dust, hygroscopicity/caking, storage stability, non-uniform dissolution, and rapid setting in Ca-rich systems	One-part denotes the preparation route, not binder chemistry; it is not water-free or risk-free
CCR/CS-assisted waste-derived binders—FA–CCR, CG–CCR, glass–CCR, GGBFS–CS, CCR–silica fume [7,70,91,92,99,123]	CCR/CS supplies Ca and OH^−^ and may act as activator, co-activator, or Ca source	Depending on formulation: N-A-S-H + C-(A)-S-H, or mainly C-S-H/C-(A)-S-H, C-A-H/AFm, and carbonates	Moderate CCR/CS can accelerate reaction; excessive dosage may increase viscosity, reduce the reactive-precursor fraction, or leave unreacted Ca-rich phases	Can reduce commercial-alkali demand and enable high waste utilization; demonstrated in several soft-clay systems	Strong dosage and source dependence; possible volume instability, high alkalinity, and leaching concerns	Includes hybrid systems and hydration/pozzolanic binders; identify the actual role of CCR/CS
Soda/alkali-residue cementitious systems—alkali residue with steel slag, GGBFS, or related solids [29,124,125]	Residue supplies alkalinity and Ca-bearing salts, often combined with reactive Ca-/Si-rich solids	C-S-H/C-(A)-S-H, AFt, and other salt-bearing phases depending on formulation	Can react readily at ambient temperature, but response is strongly soil- and formulation-dependent	High local-waste utilization and reduced demand for conventional binder; marine-soft-soil applications have been demonstrated	Source variability, chloride/salt migration, efflorescence, leaching, and possible expansive products require long-term verification	Usually better described as waste-derived cementitious or alkali-activated binders; do not label as geopolymer unless geopolymerization is demonstrated

Note: FA = fly ash; GGBFS = ground-granulated blast-furnace slag; MK = metakaolin; CCR = calcium carbide residue; CG = coal gangue; CS = carbide slag. Reaction products are reported or cautiously synthesized from the cited evidence; XRD and SEM–EDS alone do not uniquely identify amorphous gels. “One-part” denotes the preparation route rather than a distinct binder chemistry. Dosage comparisons require specifying both the material (precursor, dry activator, activator solution, or grout) and the mass denominator (dry soil, wet soil, total solids, or precursor). NaOH solution molarity should not be equated with final pore-solution concentration. Total mixture water includes soil water, added water, and water in activator solutions.

**Table 3:** Synthesis of engineering performance, governing factors, conditional findings, and design implications.

Performance Domain	General Trend After Treatment	Dominant Controls	Conditional/Contrasting Findings	Evidence Coverage	Engineering Implication
Fresh-state behavior	Workability and setting are highly sensitive to water content, precursor reactivity, and activator chemistry; added water generally improves fluidity and extends setting	Total water; FA/GGBFS ratio; activator type, modulus, and concentration; precursor fineness; temperature; soil plasticity	Higher water content can improve workability but may dilute the activator and reduce strength; the maximum-UCS mixture may not provide a practical processing window [35,120]	Very sparse (2)	Define acceptable workability, setting time, and strength together rather than optimizing UCS alone
UCS	Generally increases with effective binder dosage and curing age	Total water; binder dosage and reactivity; calcium regime; activator chemistry; curing time, temperature, and stress; soil mineralogy and organic matter	Low-Ca FA may gain strength slowly but continue developing at later ages, whereas Ca-/GGBFS-rich systems generally harden faster; optimum dosage and activator proportions shift with soil water state [5,6,69,70,119,120,123]	Rich (63)	Specify target UCS at the relevant curing age and environmental condition; do not transfer numerical optima between soils or formulations
Stress–strain/ stiffness	Stiffness generally increases with cementation, whereas deformation capacity and post-peak behavior vary	Bond density; porosity; binder content; soil type; confinement; curing stress; fibers	Higher strength does not necessarily imply greater ductility; stronger cementation may change strain-hardening behavior to pronounced strain softening, and stiffness–UCS relationships are system-specific [31,69,72,80,92]	Rich (31)	Report the full stress–strain response, stiffness, failure strain, and post-peak behavior rather than UCS alone
Shear/triaxial	Peak shear strength and apparent cohesion generally increase	Binder content and bond density; confining stress; drainage and stress path; curing; soil fabric and mineralogy	Cohesion commonly increases strongly, whereas friction angle may increase, remain similar, or decrease; stronger bonding can intensify post-peak softening [4,80,92]	Moderate (11)	Use service-relevant stress paths and confinement; report total- and effective-stress parameters, peak and residual strength, and failure mode
Compressibility/ settlement	Compressibility generally decreases, while yield stress and constrained modulus increase	Initial void ratio/water content; binder content; curing stress; filler or sand fraction; applied stress relative to structural yield	High-water-content treated soil may retain appreciable compressibility despite adequate strength; bond destructuration can increase post-yield compression, while filler effects may be primarily physical rather than chemical [4,31,92,122,139]	Moderate (11)	Conduct consolidation tests across the field-relevant stress range and report yield stress; strength alone is insufficient for deformation-based design
Permeability/ consolidation	Hydraulic conductivity often decreases through pore filling and reduced connectivity, but the trend is not universal	Pore connectivity; void ratio; cracking; binder dosage; curing; stress level; drainage condition	Some treated soils exhibit higher hydraulic conductivity or faster consolidation than cement-treated soil; this may benefit drainage but is undesirable for barriers [1,4,69,135]	Moderate (9)	Set application-specific hydraulic targets; distinguish barrier/seepage-control from drainage/consolidation functions and evaluate stress-dependent hydraulic conductivity where relevant
Tensile/flexural	Tensile and flexural resistance generally improve, particularly with fiber reinforcement	Bond continuity; fiber content, length, and dispersion; interface quality; porosity and cracking	Compressive-strength gains do not translate proportionally into tensile resistance; excessive fiber can introduce defects, and indirect or flexural tests do not directly represent uniaxial tensile strength [28,30,126]	Limited (3)	For cracking-sensitive applications, measure tensile/flexural behavior, ductility, and preferably fracture energy rather than infer them from UCS
CBR/resilient modulus	Pavement-oriented studies generally report improved bearing response	Moisture and soaking; compaction density; binder chemistry; curing; swell potential	Evidence is dominated by expansive, high-plasticity, and subgrade soils rather than core soft clays; in some studies, soaked CBR was lower than cement-treated values or decreased at later ages despite satisfying specification requirements [112,144]	Moderate (7)	Use soaked CBR, resilient modulus, swell, and deformation criteria for pavement design; very high UCS is not an adequate substitute for pavement-specific performance
Cyclic/dynamic	Evidence is too sparse to establish a robust general trend	FA/GGBFS ratio; curing stress and time; confining pressure; cyclic-stress ratio and frequency	Studies cover soft-clay cyclic response [145] and granite residual-soil impact resistance [127], but soft-soil fatigue and repeated-load data remain scarce.	Very sparse (2)	Require cyclic or dynamic testing where traffic or seismic loading governs; avoid extrapolation from monotonic tests

Note: Evidence counts correspond to the laboratory/mixture-scale evidence in Figure 3. Evidence coverage follows Figure 3 (Rich ≥ 20; Moderate = 6–19; Limited = 3–5; Very sparse = 1–2) and represents the breadth of eligible original studies rather than methodological quality or confidence. Representative references summarize within-study trends rather than a normalized cross-study ranking. Comparisons require accounting for soil water content and density, dosage basis, curing conditions and age, test method, and stress/strain level. UCS, triaxial strength, and different stiffness measures are not interchangeable. CBR/resilient-modulus evidence is derived largely from related expansive, high-plasticity, and subgrade soils rather than directly from soft clays.

**Table 4:** Capabilities and evidential limits of analytical techniques used to interpret reaction products and stabilization mechanisms.

Technique/Representative References	Information Provided	Claims Reasonably Supported	Claims Not Supported Alone	Complementary Methods	Use in Reviewed Literature/Critical Note
XRD [9,33,34]	Crystalline phases, residual minerals, and diffuse scattering associated with disordered/amorphous material	Identification and evolution of crystalline phases; qualitative evidence of changes in amorphous reaction products	Unique identification or quantification of N-A-S-H versus C-(A)-S-H; reaction degree from peak-intensity changes alone	FTIR/TG + MAS NMR; quantitative XRD with appropriate internal-standard methods	Very common. Reliable for crystalline phases, but amorphous humps are non-specific and are often overinterpreted
SEM–EDS [5,6,35]	Morphology, interfaces, cracks, and local elemental composition	Particle coating and bridging, densification, crack development, local elemental enrichment, and qualitative interface evolution	Chemical bonding; unique identification of N-A-S-H, C-(A)-S-H, or hybrid gels; bulk phase fractions	XRD + FTIR/NMR; advanced microscopy or spectroscopy where required	Very common. Local EDS composition should not be treated as unique evidence of gel identity
FTIR [5,91]	Vibrational environments and shifts in Si–O–T, carbonate, and hydroxyl bands	Changes in aluminosilicate bonding environments and qualitative reaction or polymerization trends	Unique gel identity, quantitative phase fractions, or spatial distribution	MAS NMR + XRD/TG	Used selectively. Useful as supporting evidence, but overlapping bands limit unique assignments
TG–DTG/DSC [34,35,138]	Mass-loss and thermal events associated with dehydration, dehydroxylation, and carbonate decomposition	Relative changes in bound water, carbonates, portlandite, and selected hydrates where thermal events can be distinguished	Unique attribution of overlapping events to N-A-S-H or C-(A)-S-H; nanoscale gel structure	XRD + FTIR/NMR	Used selectively. Most reliable when interpreted comparatively because thermal events commonly overlap
^29^Si/^27^Al MAS NMR [42,121]	Q^n^(mAl) environments, Al coordination, and network polymerization	Network connectivity, coordination changes, and Si/Al-dependent structural evolution	Spatial distribution of gels, interfaces, pores, or field-scale heterogeneity	XRD + SEM–EDS + pore characterization	Rare; direct structural evidence exists for kaolin stabilized with alkali-activated high-calcium fly ash, with additional evidence from non-soil binder studies. Provides stronger structural evidence for amorphous reaction products than XRD or SEM–EDS alone.
MIP/N_2_ adsorption [35,42,92,123]	Accessible pore-throat or pore-size distributions, pore volume, and gas-accessible surface area	Pore refinement and changes in accessible pore populations with binder dosage or curing	True three-dimensional connectivity, undisturbed crack architecture, or chemical identity of binding products	X-ray CT + SEM; chemical or spectroscopic characterization	Moderately used. Accessible size ranges and drying, intrusion, or adsorption assumptions must be considered when relating pore structure to performance
X-ray CT (micro-CT) [31,145]	Three-dimensional pore/crack geometry and connectivity above the effective spatial resolution	Three-dimensional heterogeneity, macro-defect evolution, and connectivity of resolvable pores and cracks	Nanoscale pores, gel chemistry, or molecular-scale interfaces	MIP/N_2_ adsorption + SEM–EDS + spectroscopy	Rare. Direct soft-soil applications exist, but resolution, segmentation, and small scanned volumes limit representativeness
Advanced probes: XAS, total-scattering PDF, STEM–EELS/HRTEM	Local coordination, short-range order, and nanoscale phase or interface information, depending on the technique	Higher-confidence structural and interfacial assignments for poorly crystalline or amorphous reaction products	Representative bulk phase fractions or field-scale heterogeneity when used alone	MAS NMR + XRD + multiscale microscopy and pore characterization	Major evidence gap. No clear direct application was identified among the reviewed soft-soil studies; targeted use could strengthen future gel and interface assignments

Note: XRD = X-ray diffraction; SEM–EDS = scanning electron microscopy with energy-dispersive X-ray spectroscopy; FTIR = Fourier-transform infrared spectroscopy; TG/DTG = thermogravimetry/derivative thermogravimetry; DSC = differential scanning calorimetry; MAS NMR = magic-angle-spinning nuclear magnetic resonance; MIP = mercury intrusion porosimetry; CT = computed tomography; XAS = X-ray absorption spectroscopy; PDF = pair distribution function; STEM–EELS = scanning transmission electron microscopy with electron energy-loss spectroscopy; HRTEM = high-resolution transmission electron microscopy. References denote representative studies using the listed techniques; complementary methods were not necessarily applied in those studies.

**Table 5:** Application readiness and remaining validation needs for geopolymer and related alternative binders in soil stabilization.

Application	Representative Systems/Evidence	Key Performance Requirements	Current Evidence Level	Main Barriers to Broader Application	Required Validation
Shallow stabilization/ stabilized (sub)base layers	MK/FA-based geopolymer stabilization and full-scale alkali-activated FA trial [141,160]	Workability; mixing and compaction; strength and stiffness; deformation; water stability	Field-demonstrated evidence	Moisture control; liquid-activator handling and transport; construction variability; cost and embodied impacts	Multi-site field sections; seasonal durability; spatial QC; long-term deformation; performance-based cost and LCA
Deep soil mixing (DSM)-oriented stabilization	FA–GGBFS and low-Ca FA systems; one-part solid activation [30,31,35,69,71]	Binder deliverability; working and setting time; column strength and stiffness; permeability; spatial uniformity	Validated laboratory performance	Scale-dependent mixing; binder delivery; radial and vertical variability; stress-dependent curing; groundwater chemistry	Full-scale DSM columns; coring and in situ tests; mixing and delivery control; strength and permeability variability; long-term performance
Road subgrades, embankments, and engineered fill	Alkali residue, CCR, CS–CG, SS–RHA, and ternary waste binders [47,48,111,143,144,158]	CBR and resilient response; compaction; swelling; durability; settlement; environmental safety	Pilot/field evidence †	Soil and waste variability; coupled traffic–moisture–climate effects; leaching risk; construction QC	Repeated-load testing; seasonal field monitoring; coupled durability; groundwater/leachate monitoring; performance-based acceptance criteria
Dredged-soil reuse/ high-water-content engineered fill	GGBFS–FA–DG and related waste-derived binders [1,3]; Fluorogypsum–calcined shell powder [161].	Flow and mixability; strength; compressibility; permeability; volume stability; leaching	Laboratory performance and small-scale outdoor tests.	High and variable water content; pore-water chemistry; large-volume mixing; spatial nonuniformity	Large-batch or pilot placement; field mixing and pumpability; settlement and permeability monitoring; leaching and spatial QC
Hydraulic barriers/ seepage control	Low-Ca FA and ternary waste-binder-treated soils [1,69]	Low and stable hydraulic conductivity; crack resistance; chemical durability; adequate strength	Laboratory proof	Low hydraulic conductivity is not universally achieved; strength and hydraulic conductivity do not necessarily correlate; stress, cracking, and chemical exposure may create preferential flow paths	Stress-dependent saturated hydraulic conductivity; coupled wetting–drying, chemical, and cracking tests; large-scale barrier continuity; long-term transport and leaching
Grouting/jet-grouting-type applications	Alkali-activated FA soft-soil mixtures and geopolymer grouts [120,134,165]	Rheology and viscosity; injectability; setting time; bleeding and segregation; strength; soil–grout interaction	Field-demonstrated evidence ‡	High viscosity; filtration and penetrability; short working time; activator delivery; uncertain in situ uniformity	Injection or jet-grouting trials; rheology–injectability–strength optimization; full-scale treated columns; permeability and environmental monitoring

Note: Evidence level denotes the highest application-specific validation identified in the reviewed literature: laboratory proof, validated laboratory performance, pilot/field evidence, or field-demonstrated evidence. Field-demonstrated evidence refers to successful implementation of at least one specific binder–soil–construction system and does not imply general field readiness, code qualification, or transferability to soft-soil applications. † Field evidence in this category partly derives from related high-plasticity and subgrade soils and from waste-derived binder systems rather than core soft-clay geopolymer systems. ‡ Evidence is limited to the specific jet-grouting application reported by Cristelo et al. [134]; transferability to other soils and construction conditions requires further verification. Abbreviations: FA = fly ash; GGBFS = ground-granulated blast-furnace slag; MK = metakaolin; CCR = calcium carbide residue; CS = carbide slag; CG = coal gangue; SS = steel slag; RHA = rice husk ash; DG = desulfurization gypsum; DSM = deep soil mixing; QC = quality control; LCA = life-cycle assessment.

## Data Availability

No new data were created or analyzed in this study. Data sharing is not applicable to this article.

## Supplementary Materials

The following supporting information can be downloaded at: https://www.mdpi.com/article/10.3390/ma19214546/s1, Table S1: Study-level classifications and category totals for the original publications included in the evidence map.
