# Supplementary material for: Geopolymer, Alkali-Activated, and Waste-Derived Binders for Soft-Soil Stabilization: A Critical Review
Source: Materials (Basel). 2026 Oct 28;19(21):4546. doi: 10.3390/ma19214546 (PMC13764841; doi:10.3390/ma19214546)
Supplement: Supplementary file 1 [file materials-19-04546-s001.zip › materials-4641482-supplementary.pdf]

Supplementary Table S1

Geopolymer, Alkali-Activated, and Waste-Derived Binders for Soft-Soil Stabilization: A Critical Review

Study-level classification of the 74 original publications included in the evidence map. Reference links lead to the source DOIs; full bibliographic details are given in the main article.

Counting. Each publication is counted once per reported performance domain and scale. Mixtures, specimens and curing ages do not create additional counts. Multiple domains and scales may overlap. Reviews and neat-binder tests are excluded; related soil-based materials are identified explicitly.

Soils. C = core soil group (soft, marine/dredged or model-clay sources); R = related soils or soil-based materials; C/R = both. These are scope categories, not certainty ratings. Tested preparation is retained: a C label alone does not establish direct evidence for saturated, high-water-content soft clay.

Binders. Entries describe tested formulations, not confirmed gel structures. Conventional controls are omitted unless needed to interpret the tests. FA = fly ash; GGBFS = ground granulated blast-furnace slag; GBFS = granulated blast-furnace slag; CCR = calcium carbide residue (including carbide slag/sludge); MK = metakaolin; CG = coal gangue; RHA = rice husk ash; BOFS = basic oxygen furnace slag; OPC = Portland cement. “Silicate” denotes sodium silicate unless stated otherwise.

Evidence. L = laboratory or mixture-scale assessment; FT = field trial/project assessment; FM = instrumented field monitoring. FRESH = fresh-state behavior; UCS = unconfined compressive strength; SS = stress–strain/stiffness; SH = shear/triaxial; CS = compressibility/settlement; PERM = hydraulic conductivity; TF = tensile/flexural; CBR = California bearing ratio/resilient modulus; CYC = cyclic/dynamic; PD = physical durability; CD = chemical durability; ENV = environmental compatibility/life-cycle assessment (LCA); SL = service-life prediction. UU = unconsolidated-undrained triaxial test.

Reference	Soil group and tested material	Binder system(s)	Evidence domain(s) and scale
**S01** Abdullah et al. (2019)	R: Compacted kaolin and two natural clay soils	FA–GGBFS; NaOH/silicate	L: UCS, SS, SH
**S02** Arulrajah et al. (2018)	C: High-water-content marine clay (Coode Island Silt)	FA–GGBFS; NaOH/silicate	L: UCS, SS, TF
**S03** Bazarbekova et al. (2024)	R: Compacted natural clay at three initial moisture contents	Class C/F FA or FA–GGBFS; with/without sodium metasilicate	L: UCS
**S04** Chen et al. (2020)	C: Soft-plastic natural soil	FA–GGBFS–OPC; NaOH/silicate	L: UCS, CS, PERM
**S05** Chen et al. (2023)	C: Soft clay reconstituted at 1.0 and 1.4 liquid limits	Low-calcium FA; NaOH/silicate	L: UCS, SS, PERM
**S06** Cristelo et al. (2011)	C: Sandy lean clay/slurry and jet-grouted soil columns	Low-calcium FA; NaOH/silicate	L: UCS, SS<br>FT: UCS
**S07** Cristelo et al. (2012)	C: Marl-derived high-plasticity clay; cast slurry	Class C and Class F FA; NaOH/silicate	L: UCS
**S08** Cristelo et al. (2013)	C: Sandy lean clay mixed as a high-water-content slurry	Low-calcium FA; NaOH/silicate	L: UCS
**S09** Deng et al. (2025)	C: Excavated waste mud treated as a soil slurry	GGBFS–steel slag–desulfurization gypsum	L: UCS, PERM
**S10** Du et al. (2017)	R: Foamed lightweight material containing low-plasticity clay	GGBFS–CCR–silicate; preformed foam	L: UCS, PERM
**S11** Du et al. (2025)	C: Marine soft clay at varied high water contents	CCR–FA; NaOH	L: UCS
**S12** Gaddam and Yamsani (2024)	R: Compacted expansive black cotton soil	Coal gangue ± GGBFS; NaOH/silicate	L: UCS, CBR, PD

Reference	Soil group and tested material	Binder system(s)	Evidence domain(s) and scale
**S13** Ghadir and Ranjbar (2018)	R: Compacted low-plasticity clay	Volcanic ash; NaOH	L: UCS, SS
**S14** Ghadir et al. (2021)	R: Compacted low-plasticity clay	Volcanic ash; NaOH	L: SH, ENV
**S15** Horpibulsuk et al. (2012)	R: Compacted problematic silty clay	CCR ± FA	L: UCS
**S16** Horpibulsuk et al. (2013)	R: Compacted silty clay	CCR ± FA	L: UCS, PD
**S17** Hua et al. (2026)	R: Road soil treated with alkali residue	Desalinated alkali (soda) residue	L: CBR, PD, ENV
**S18** Jia and Chu (2025b)	C: Reconstituted Tianjin soft clay	Low-calcium FA; NaOH/silicate	L: SS, SH
**S19** Kampala and Horpibulsuk (2013)	R: Compacted high-plasticity silty clay	CCR	L: UCS, CS, CBR, PD
**S20** Kampala et al. (2013)	R: Recycled calcium-carbide-residue-stabilized clay	Recycled CCR-stabilized clay	L: UCS, SS, CS, PD
**S21** Lazorenko and Kasprzhitskii (2026)	C/R: Model mineral/organic soils, including peat-rich mixtures	MK; NaOH/silicate	L: UCS
**S22** Lei et al. (2026)	C: Artificially structured clay prepared from Tianjin clay	MK; NaOH/silicate	L: SS, SH
**S23** Li et al. (2021)	C: High-water-content marine soft clay	Coal gangue–CCR; NaOH/silicate	L: SS, SH
**S24** Li et al. (2023)	C: Marine soft clay	Coal gangue–CCR; NaOH/silicate	L: SS, SH, CS
**S25** Li et al. (2024a)	C: Soft clay at natural high water content	FA–GGBFS; solid NaOH	L: UCS, CD
**S26** Liu et al. (2019)	R: Compacted expansive soil	RHA–CCR	L: UCS, SH
**S27** Liu et al. (2024)	R: Compacted subgrade soil	Calcined CG–CCR	L: UCS, CBR, PERM, PD, CD, ENV
**S28** Liu et al. (2026)	C: High-water-content Shanghai dredged slurry	Cement–steel slag–GGBFS–quicklime	L: FRESH, CS, PERM
**S29** Maheepala et al. (2024)	R: Compacted expansive clay	FA; NaOH + RHA-derived silicate	L: UCS, CBR, ENV
**S30** Maheepala et al. (2026)	R: Compacted expansive clay	FA–GGBFS; NaOH + RHA-derived silicate	L: UCS, CBR, PD, ENV
**S31** Miao et al. (2017)	R: Compacted expansive black cotton soil	Volcanic ash; KOH or $Ca(OH)_2$	L: UCS
**S32** Min et al. (2021)	C: High-water-content soft clay slurry	GGBFS ± FA; solid NaOH	L: UCS, SS, CS
**S33** Min et al. (2022)	C: High-water-content soft silty clay	GGBFS ± FA; solid NaOH	L: CYC
**S34** Min et al. (2023)	C: High-water-content soft clay slurry	GGBFS ± FA; solid silicate	L: UCS

Reference	Soil group and tested material	Binder system(s)	Evidence domain(s) and scale
**S35** Miraki et al. (2022)	R: Compacted low-plasticity clay	Volcanic ash–GGBFS; NaOH	L: UCS, PD, ENV
**S36** Miranda et al. (2020)	R: Compacted sandy lean clay in pavement subbase	FA; NaOH ± silicate or recycled alkaline solution	L: UCS, SS<br>FT: UCS, SS, ENV
**S37** Nagaraju et al. (2023)	R: Compacted expansive clay	FA or GGBFS; NaOH/silicate	L: UCS, SS, ENV
**S38** Nalbantoglu and Gucbilmez (2001)	R: Compacted calcareous expansive soil	High-calcium FA ± lime	L: CS, PERM
**S39** Odeh and Al-Rkaby (2022)	R: Compacted clay with varied sand contents	High-calcium FA; NaOH/silicate	L: UCS, SS, CD
**S40** Phetchuay et al. (2014)	R: Compacted problematic high-plasticity silty clay	High-calcium FA–CCR; silicate	L: UCS, PD
**S41** Phetchuay et al. (2016)	C: High-water-content Coode Island Silt	FA ± CCR; NaOH/silicate	L: UCS, ENV
**S42** Pourabbas Bilondi et al. (2018)	R: Compacted low-plasticity clay	Recycled glass powder–CCR	L: UCS, SS
**S43** Qin et al. (2026a)	C: High-water-content soft-clay slurry with low sand additions	CCR-activated GGBFS; separate OPC comparator	L: FRESH, UCS, CS, PERM
**S44** Qin et al. (2026b)	C: Marine soft clay	GGBFS ± biomass FA; CCR/phosphogypsum	L: UCS, SS, TF, PD, ENV
**S45** Sahoo and Singh (2022)	R: Compacted expansive clay	GGBFS; NaOH	L: UCS, SS, PD
**S46** Salimi and Ghorbani (2020)	C: Kaolinite model clay; compacted at optimum water content	GBFS or BOFS; lime/MgO ± NaOH/silicate	L: UCS, SS, CS
**S47** Samuel et al. (2020)	R: Compacted expansive high-plasticity clay	MK; KOH + silica fume	L: UCS, ENV
**S48** Shang et al. (2023)	R: Compacted mica-schist-weathered subgrade soil	Cement–FA–GGBFS–desulfurization gypsum	L: UCS, SS, SH
**S49** Su et al. (2023)	C: Soft clay with controlled humic-acid contents	FA–GGBFS; NaOH/silicate	L: UCS, SS
**S50** Sukmak et al. (2013)	R: Compacted clay–FA bricks; heat-cured (supporting material)	FA; NaOH/silicate	L: UCS
**S51** Tang et al. (2025)	C/R: Kaolin at liquid-limit and lower water contents	CCR ± FA; with/without glass-derived silicate	L: UCS
**S52** Tang et al. (2026)	C: Kaolin at liquid limit; statically compacted	CCR ± FA; with/without glass-derived silicate	L: UCS, PD, ENV
**S53** Wang et al. (2021b)	R: Compacted clay reinforced with basalt fibres	MK; CaO/silicate; optional basalt fibres	L: UCS, SS, SH, TF
**S54** Wang et al. (2022a)	C: Soft clay slurry at 50% water content	GGBFS ± FA; NaOH/silicate	L: UCS, CD

Reference	Soil group and tested material	Binder system(s)	Evidence domain(s) and scale
**S55** Wang et al. (2022c)	C: Marine-origin clay; compacted mixtures	Soda residue–steel slag–GGBFS	L: UCS, SS
**S56** Wang et al. (2023a)	C: Dredged clay at 1.4-2.0 liquid limits	FA–GGBFS–gypsum; NaOH/silicate	L: UCS
**S57** Wassie and Demir (2024)	C: Soft-deposit silt; reconstituted/compacted at 0.75 liquid limit	MK–OPC (UCS); NaOH/silicate-activated MK (UU)	L: UCS, SS, SH
**S58** Wei et al. (2026)	R: Red clay, sandy soil and loessial silt; flowable molded mixtures	Soda residue with activated GGBFS or OPC–GGBFS	L: UCS, PD
**S59** Wu et al. (2022)	C: High-water-content Hangzhou silty soft clay	FA–GGBFS; NaOH/silicate	L: UCS, PD
**S60** Yaghoubi et al. (2018)	C: High-water-content Coode Island Silt	FA, FA–GGBFS or GGBFS; Na/K hydroxides/silicates	L: UCS, SS
**S61** Yaghoubi et al. (2019)	C: High-water-content Coode Island Silt	FA–GGBFS; NaOH/silicate	L: UCS, PD
**S62** Yaghoubi et al. (2022)	C: High-water-content Coode Island Silt	FA–GGBFS; NaOH/silicate	L: UCS, SS, SH, CS, PERM, ENV
**S63** Yang et al. (2022)	C: Marine-origin silt; dried and compacted at 16% water content	CCR–plant ash	L: UCS, PD
**S64** Yi et al. (2015)	C: Marine soft clay	CCR-activated GGBFS	L: UCS
**S65** Shao et al. (2018)	C: Marine-origin soil reconstituted by compaction	Alkali residue (soda-industry residue)	L: UCS, SS, PD
**S66** Yu et al. (2026)	R: High-plasticity expansive highway subgrade clay	Steel slag–rice husk ash	L: CS, CBR<br>FT: CS<br>FM: CS
**S67** Yuan et al. (2022)	R: Compacted granite residual soil with optional fibres	MK + NaOH/silicate; optional basalt/glass fibres	L: UCS, SS, CYC
**S68** Zhang et al. (2013a)	R: Compacted synthetic lean clay	MK; NaOH/silicate	L: UCS, SS
**S69** Zhang et al. (2015)	R: Compacted synthetic sulfate-rich lean clay	MK; NaOH/silicate	L: UCS, SS, CD
**S70** Zhou et al. (2021)	C: Marine soft clay reconstituted at high water contents	Coal-derived MK or GGBFS; NaOH/silicate	L: UCS
**S71** Zhou et al. (2026)	R: Silt, loess, sands and gravel; compacted or vibrated	FA, GGBFS or MK; solid NaOH/silicate	L: UCS, SS
**S72** Zhu et al. (2024)	C: Dredged sludge at natural water content	Steel slag–FA–CCR	L: UCS, ENV
**S73** Zhu et al. (2025b)	C: Marine clay with adjusted water and humic-acid contents	CCR–silica fume	L: UCS, ENV
**S74** Gu et al. (2026)	C: Dredged sludge prepared at 100% water content	Fluorogypsum–calcined shell powder (4:1); 15–25% by dry soil mass	L: SS, PD, ENV

Category totals

Soil groups: C = 38; R = 34; C/R = 2 (74 publications in total).

Binder constituents (non-exclusive): FA-containing = 38; blast-furnace-slag-containing = 32; CCR-containing = 21; publications containing none of these three constituents = 15. FA includes the biomass fly ash in S44. These counts concern experimental binder constituents, not mutually exclusive reaction pathways; their sum is not a publication total.

Laboratory/mixture-scale domains: FRESH = 2; UCS = 63; SS = 31; SH = 11; CS = 11; PERM = 9; TF = 3; CBR = 7; CYC = 2; PD = 18; CD = 5; ENV = 15; SL = 0.

Field trials/project assessments: UCS = 2; SS = 1; CS = 1; ENV = 1; all other domains = 0. Field monitoring: CS = 1; all other domains = 0. No large-scale physical-model or service-life-prediction study is assigned. The FT and FM entries for S66 describe the same field campaign, with approximately 150 days of settlement monitoring.

Coding qualifications

Codes identify separately reported outcomes, not every measurement used in preparation or calculation. In S10, flowability is a preparation target; in S17, UCS underlies a water-stability index without a separate original absolute-UCS result. Confined triaxial strength is not UCS. Dynamic-only response is assigned to CYC; SS requires separate monotonic stress–strain or stiffness evidence. PERM includes direct and consolidation-derived values. Water absorption or drying shrinkage alone is not coded as durability. Environmental assessments include leaching and original carbon/LCA inventories, but not general sustainability statements or cost alone.

S50 concerns heat-cured clay–FA bricks and supplies indirect evidence from a related soil-based material. In S57, UCS concerns MK–OPC blends, whereas activated MK was examined in UU tests. Publication-level outcome codes therefore must not be attributed to every formulation within a paper. Counts indicate coverage, not study quality, statistical independence or confidence.

For S74, SS denotes the reported compressive modulus. Bearing capacity was not equated with UCS, and tests mentioned without separately identifiable results were not counted. The small-scale outdoor setup was treated as mixture-scale evidence rather than a project-scale field trial or instrumented monitoring.
